# Integrative single-cell and bulk transcriptomics uncovers a lipid metabolism-related lncRNA signature alongside the ELFN1-AS1/LYPLA1 axis driving osteosarcoma progression

**DOI:** 10.3389/fimmu.2026.1945005

**Published:** 2026-09-11

**Authors:** Xianfu Wei, Zhipeng Wang, Qiang Yang, Feiyue Lin, Long Chen

**Affiliations:** 1 Department of Orthopedics, Qilu Hospital of Shandong University, Jinan, Shandong, China; 2 Department of Musculoskeletal Oncology, Clinical Oncology School of Fujian Medical University, Fujian Cancer Hospital, Fuzhou, China

**Keywords:** ELFN1-AS1/LYPLA1 axis, lipid metabolism, lncRNA, osteosarcoma, prognostic signature, tumor immune microenvironment

## Abstract

**Background:**

Osteosarcoma is the most prevalent primary malignant bone tumor in adolescents, featuring prominent intratumoral heterogeneity, early pulmonary metastasis and stagnant survival improvement for metastatic/chemoresistant patients. Altered lipid metabolism represents as a core adaptive hallmark of malignancy, whereas the prognostic implication and molecular regulatory functions of lipid metabolism-related long non-coding RNAs (LMRLs) remain poorly elucidated in osteosarcoma.

**Methods:**

Single-cell RNA-seq data from GSE152048 and bulk transcriptomic and clinical data from TARGET-OS were integrated. Lipid metabolic activity, malignant cell-state transitions, and intercellular communication were evaluated using AUCell, Monocle, and CellChat, respectively. Lipid metabolism-related genes were identified by integrating single-cell differential expression analysis with WGCNA. A prognostic lncRNA signature was constructed using Pearson correlation, Cox regression, and LASSO analyses. Immune infiltration, tumor mutational burden, immune checkpoint expression, and drug sensitivity were further assessed. scTenifoldKnk-based virtual knockout screening was performed to predict downstream target genes of ELFN1-AS1, followed by comprehensive *in vitro* and *in vivo* functional validation of the ELFN1-AS1/LYPLA1 regulatory axis.

**Results:**

Single-cell analysis revealed marked lipid-metabolic heterogeneity across osteosarcoma cell populations, with dynamic remodeling along malignant cell-state transitions and enhanced communication between high-lipid-metabolism osteosarcoma cells and stromal or vascular compartments. By integrating single-cell and bulk transcriptomic evidence, 22 lipid metabolism-related candidate genes were identified, from which a five-LMRL signature comprising AL133410.1, AL596247.1, ELFN1-AS1, IL10RB-DT, and NECTIN3-AS1 was developed. This signature effectively stratified patients into prognostically distinct risk groups and remained an independent predictor of overall survival. Exploratory computational analyses indicated risk subgroups exhibited divergent immune landscapes, mutational profiles and predicted drug responsiveness. Virtual knockout screening prioritized LYPLA1 as the key downstream effector of ELFN1-AS1. Among the signature lncRNAs, ELFN1-AS1 was prominently upregulated in osteosarcoma tissues and cells and was associated with poor prognosis. Mechanistically, ELFN1-AS1 drives osteosarcoma progression by triggering LYPLA1-dependent lipid accumulation-associated phenotype. Pharmacological blockade of *de-novo* lipogenesis using orlistat (selective FASN inhibitor) attenuated ELFN1-AS1-driven oncogenic phenotypes.

**Conclusion:**

This study establishes an LMRL-based for osteosarcoma prognostic stratification that reflects immune, genomic and therapeutic heterogeneity. Combining virtual knockout screening and experimental validation, we characterize the oncogenic ELFN1-AS1/LYPLA1 regulatory cascade as a potential biomarker and therapeutic target for precision management of osteosarcoma.

## Introduction

Osteosarcoma (OS) is the most common primary malignant bone tumor in children and adolescents, with an annual incidence of approximately 3–5 cases per million and a predilection for the metaphyseal regions of rapidly growing long bones (1, 2). The current standard treatment consists of multi-agent neoadjuvant chemotherapy, complete surgical resection, and postoperative chemotherapy, which has improved the 5-year survival rate of patients with localized disease to approximately 60%-70%. However, this survival benefit has plateaued over the past several decades, and the prognosis of patients with metastatic, recurrent, or chemotherapy-refractory disease remains poor, with long-term survival generally below 30% (3–5). Beyond its tendency for early pulmonary dissemination, osteosarcoma is characterized by pronounced intratumoral heterogeneity, whereby malignant cells differ in differentiation state, metastatic potential, and therapeutic response (6). This biological complexity contributes to substantial variation in clinical outcomes, even among patients with similar clinicopathological features who receive comparable treatment (7). Therefore, a deeper understanding of the molecular mechanisms that drive OS progression is urgently needed to identify reliable prognostic biomarkers and develop more effective therapeutic strategies.

Altered lipid metabolism has emerged as a major feature of malignant transformation, through which cancer cells secure energy, membrane components, and signaling molecules while adapting to nutrient deprivation and oxidative stress (8–10). Meanwhile, cholesterol, phospholipids, and sphingolipids influence membrane organization, oncogenic signaling, cell migration, and immune regulation (11). In addition, lipids also function as signaling mediators and redox regulators, through which they influence cancer proliferation, migration, therapeutic resistance, and regulated cell death (12). In osteosarcoma, lipid metabolism is broadly reprogrammed, as tumor cells exhibit enhanced lipid acquisition, *de novo* lipid biosynthesis, and fatty acid oxidation, through which they sustain membrane remodeling, energy production, and adaptation to metabolic and microenvironmental stress (13). Furthermore, dysregulated fatty acid and cholesterol metabolism further supports the formation and maintenance of cancer stem cells by sustaining their self-renewal and differentiation plasticity, thereby contributing to malignant progression (14). Accordingly, targeting perturbed lipid metabolism represents a promising therapeutic opportunity for osteosarcoma.

Long non-coding RNAs (lncRNAs), a class of transcripts longer than 200 nucleotides with limited protein-coding potential, have emerged as important regulators of gene expression at epigenetic, transcriptional, and post-transcriptional levels (15–17). By interacting with DNA, RNA, or proteins, lncRNAs participate in diverse malignant processes, including proliferation, invasion, metastasis, stemness maintenance, immune modulation, and therapeutic resistance (18–20). In osteosarcoma, aberrantly expressed lncRNAs have been increasingly linked to tumor progression and patient prognosis, suggesting their potential value as biomarkers and therapeutic targets (21, 22). Notably, lncRNAs can also reshape cancer metabolism by regulating metabolic enzymes and signaling pathways. For instance, HNF4A-AS1 regulates lipid metabolism through the METTL3/m6A/YTHDF3/DECR1 axis, thereby promoting sorafenib-induced ferroptosis and reducing drug resistance in HCC (23). Given that the clinical significance and molecular basis of LMRLs in osteosarcoma remain insufficiently defined, systematic investigation of lncRNAs linked to lipid metabolic states may help identify prognostic biomarkers and uncover regulatory mechanisms coupling lipid metabolic reprogramming to osteosarcoma progression.

In the present study, we integrated single-cell and bulk transcriptomic analyses to characterize lipid-metabolic heterogeneity and its prognostic relevance in osteosarcoma. Single-cell analysis revealed dynamic lipid metabolic remodeling across malignant cell states and lipid metabolism-associated differences in tumor-microenvironment communication. By combining single-cell-derived DEGs with WGCNA from the TARGET-OS cohort, we identified lipid metabolism-related genes (LMRGs) and constructed a five-LMRL prognostic signature, which stratified osteosarcoma patients into distinct risk groups with different survival outcomes, alongside exploratory computational differences in immune profiles, mutational characteristics and predicted drug responses. We performed scTenifoldKnk virtual knockout screening to predict downstream regulatory genes of core lncRNA ELFN1-AS1, and further experimental validation identified ELFN1-AS1 as a clinically relevant lncRNA that was highly expressed in osteosarcoma and associated with advanced disease and poor prognosis. Mechanistically, ELFN1-AS1 drives osteosarcoma progression through LYPLA1-dependent lipid accumulation-associated phenotype. Collectively, our study provides new insights into lncRNA-mediated lipid metabolic remodeling in osteosarcoma and highlights the ELFN1-AS1/LYPLA1 regulatory cascade as a potential prognostic biomarker and therapeutic target.

## Materials and methods

### Data collection

RNA-sequencing profiles and clinical data for 88 osteosarcoma patients were obtained from the TARGET database (Therapeutically Applicable Research to Generate Effective Treatments; http://ocg.cancer.gov/programs/target), of which 84 cases with complete clinical information were retained. In addition, the scRNA-seq dataset GSE152048 was downloaded from the Gene Expression Omnibus (GEO) database (https://www.ncbi.nlm.nih.gov/geo) and included 11 osteosarcoma samples generated using the 10× Genomics platform, comprising seven primary, two metastatic, and two recurrent tumors. LMRGs were collected by querying the Molecular Signatures Database (MSigDB) using the keyword “lipid metabolism” and were further supplemented with genes reported in previously published studies. After merging the resulting gene sets and removing duplicate entries using Microsoft Excel, 1,146 unique LMRGs were retained for subsequent analyses (**Supplementary Table 1**).

### Processing of sc-RNAseq data

The scRNA-seq data were processed in R using the Seurat framework. Cells were retained when they exhibited 400-6,500 detected genes and 600-50,000 unique molecular identifier counts; cells in which mitochondrial or hemoglobin-derived transcripts accounted for more than 20% or 1% of the total expression, respectively, were removed. Following quality-control filtering, 101,747 cells were available for downstream analyses. Inter-sample technical variation was mitigated using the Harmony algorithm, which integrates cells within a shared low-dimensional representation. The resulting cellular clusters were visualized using uniform manifold approximation and projection (UMAP). Cell identities were assigned through the combined evaluation of cluster-specific differentially expressed genes and established lineage markers reported in the literature.

### AUCell

Lipid metabolism activity at the single-cell level was quantified using the AUCell R package. Genes were ranked according to their expression within each cell, and enrichment of the predefined lipid metabolism-related gene set among highly expressed genes was summarized as an area under the curve (AUC) score. Higher AUC values indicated greater relative lipid metabolism activity. Osteosarcoma cells were subsequently stratified into high- and low-lipid-metabolism groups using the median AUC score as the cutoff for downstream analyses. AUCell is a rank-based approach that evaluates gene-set activity independently within each cell.

### Weighted gene co-expression network analysis

Weighted gene co-expression network analysis was performed in the TARGET-OS cohort to identify gene modules associated with lipid metabolism. Lipid metabolism activity in each sample was quantified using ssGSEA and incorporated as an external trait. A soft-thresholding power of 8 was selected to construct an unsigned scale-free co-expression network. Gene modules were identified with a minimum module size of 100 and merged at a cut height of 0.35. Module eigengenes were correlated with lipid metabolism scores using Pearson correlation analysis, and the module showing the strongest association was selected for subsequent analysis. Candidate hub genes were defined by an absolute module membership greater than 0.8 and an absolute gene significance greater than 0.3.

### Identification of LMRLs and construction of the prognostic lncRNA signature

Co-expression analysis between the overlapping genes and annotated lncRNAs in osteosarcoma was performed using the limma R package. LncRNAs meeting the criteria of ∣r∣>0.30 and *P* < 0.001 were defined as candidate LMRLs. The TARGET-OS cohort was then randomly divided at a 6:4 ratio into a training cohort and an internal testing cohort. Within the training cohort, prognostically relevant LMRLs were identified by univariable Cox regression (*P* < 0.05) and further refined using LASSO Cox regression with the glmnet R package. The lncRNAs retained by LASSO were subsequently entered into multivariable Cox regression to establish the prognostic signature. An individual risk score was calculated as the sum of the expression level of each model lncRNA multiplied by its corresponding regression coefficient. Once the model had been established in the training cohort, both the regression coefficients and the median risk-score cutoff derived from the training cohort were fixed and subsequently applied to the internal testing cohort without model refitting or cutoff recalibration. The proportional hazards assumption of the final Cox model was evaluated using scaled Schoenfeld residuals. Internal model stability was further assessed using 1,000 bootstrap resamples. Model performance was assessed separately in the training cohort, the internal testing cohort, and the entire TARGET-OS cohort.

### Virtual knockout analysis of ELFN1-AS1

The regulatory effects of ELFN1-AS1 were assessed across all OS cells using the scTenifoldKnk R package. A single-cell gene regulatory network was constructed, followed by in silico knockout of ELFN1-AS1 and comparison of the perturbed network with the original network. Downstream differentially regulated genes with an adjusted *P* < 0.05 were considered significant and subjected to functional enrichment analysis.

### Human clinical samples

A total of 32 patients with osteosarcoma who were treated at Qilu Hospital of Shandong University were enrolled in this study. One osteosarcoma tissue specimen and one matched adjacent non-tumor tissue specimen were obtained from each patient, resulting in 32 paired tumor-non-tumor samples and 64 individual tissue specimens in total. According to the presence or absence of distant metastasis, the 32 patients were further classified into a metastatic group comprising 20 patients and a non-metastatic group comprising 12 patients. The clinicopathological characteristics of the enrolled patients are summarized in **Supplementary Table 2**. The study protocol was approved by the Ethics Committee of Qilu Hospital of Shandong University, and written informed consent was obtained from all participants.

### Quantitative Real-time PCR

RNA was extracted from osteosarcoma cells and tissue specimens using TRIeasy reagent (Yeasen, Shanghai, China) and subsequently reverse-transcribed with the Hifair^®^ 1st Strand cDNA Synthesis SuperMix. RT-qPCR was performed using the Hifair^®^ III RT-qPCR SYBR Green Kit on a CFX Connect Real-Time PCR Detection System (Bio-Rad, Hercules, CA, USA). GAPDH was used as the endogenous reference, and relative RNA expression was calculated using the 2^–ΔΔCt^ method. Primer sequences are listed in **Supplementary Table 3**.

### Measurement of intracellular triglyceride and total cholesterol levels

Following the indicated treatments, osteosarcoma cells were harvested and lysed. Intracellular triglyceride and total cholesterol levels were measured using commercial assay kits (Solarbio, Beijing, China) according to the manufacturer’s instructions. Lipid concentrations were calculated from standard curves and normalized to total protein content.

### Oil red O staining

Following the indicated treatments, osteosarcoma cells were washed twice with PBS and fixed with 4% paraformaldehyde for 20 min. Intracellular neutral lipid droplets were subsequently stained using an Oil Red O staining kit (Solarbio, Beijing, China) according to the manufacturer’s protocol. After excess dye had been removed, the stained cells were examined and photographed under a light microscope.

### Animal models

Female BALB/c nude mice aged 4–5 weeks were obtained from SPF Biotechnology Co., Ltd. (Beijing, China) and housed in a specific pathogen-free facility at Qilu Hospital of Shandong University. A total of 10 mice were used, with 5 mice in each group. Before inoculation, mice were randomly allocated to the experimental groups using a computer-generated sequence. 143B cells stably expressing sh-NC or sh-ELFN1-AS1 were suspended in PBS, and 3 × 10^6^ cells in 200 μL were implanted subcutaneously into the flank of each mouse. Tumor dimensions were monitored at regular intervals with a caliper, and tumor volume was calculated as V = (length × width^2^)/2. Endpoint tumor weighing and histological quantification of pulmonary metastatic foci were performed using coded samples by investigators blinded to group allocation. Prespecified exclusion criteria included unsuccessful cell inoculation due to substantial leakage of the cell suspension, accidental death unrelated to tumor progression. Humane endpoints included a loss of more than 20% of baseline body weight, tumor ulceration or tumor growth beyond the institutionally approved size limit, persistent inability to ambulate or obtain food and water, respiratory distress, or sustained signs of pain or distress. Animals reaching any humane endpoint were euthanized immediately. 28 days after injection, the mice were euthanized with sodium pentobarbital (100 mg/kg, intraperitoneally) followed by cervical dislocation, after which subcutaneous tumors were excised, photographed, and weighed. Lung tissues were collected concurrently to evaluate pulmonary metastasis, fixed in 4% paraformaldehyde, paraffin-embedded, sectioned, and examined by hematoxylin and eosin staining, with metastatic foci quantified between the two groups. All animal procedures were approved by the Institutional Animal Care and Use Committee of Qilu Hospital of Shandong University and conducted in accordance with institutional animal-welfare guidelines.

### Statistical analysis

Statistical analyses were performed using R (version 4.3.3) and GraphPad Prism 8.0. Data from at least three independent experiments are presented as the mean ± standard deviation. Comparisons between two groups were conducted using Student’s t-test or the Mann–Whitney U test, whereas one-way ANOVA or the Kruskal-Wallis test was applied to multiple-group comparisons, as appropriate. Kaplan-Meier analysis with the log-rank test was used for survival comparisons, and correlations were evaluated by Pearson’s or Spearman’s correlation analysis. All tests were two-sided, with *P* < 0.05 considered statistically significant. Additional methodological details are provided in the **Supplementary Materials** and Methods.

## Results

### Single-cell profiling reveals lipid-metabolic heterogeneity and intercellular crosstalk in osteosarcoma

**Figure 1:**
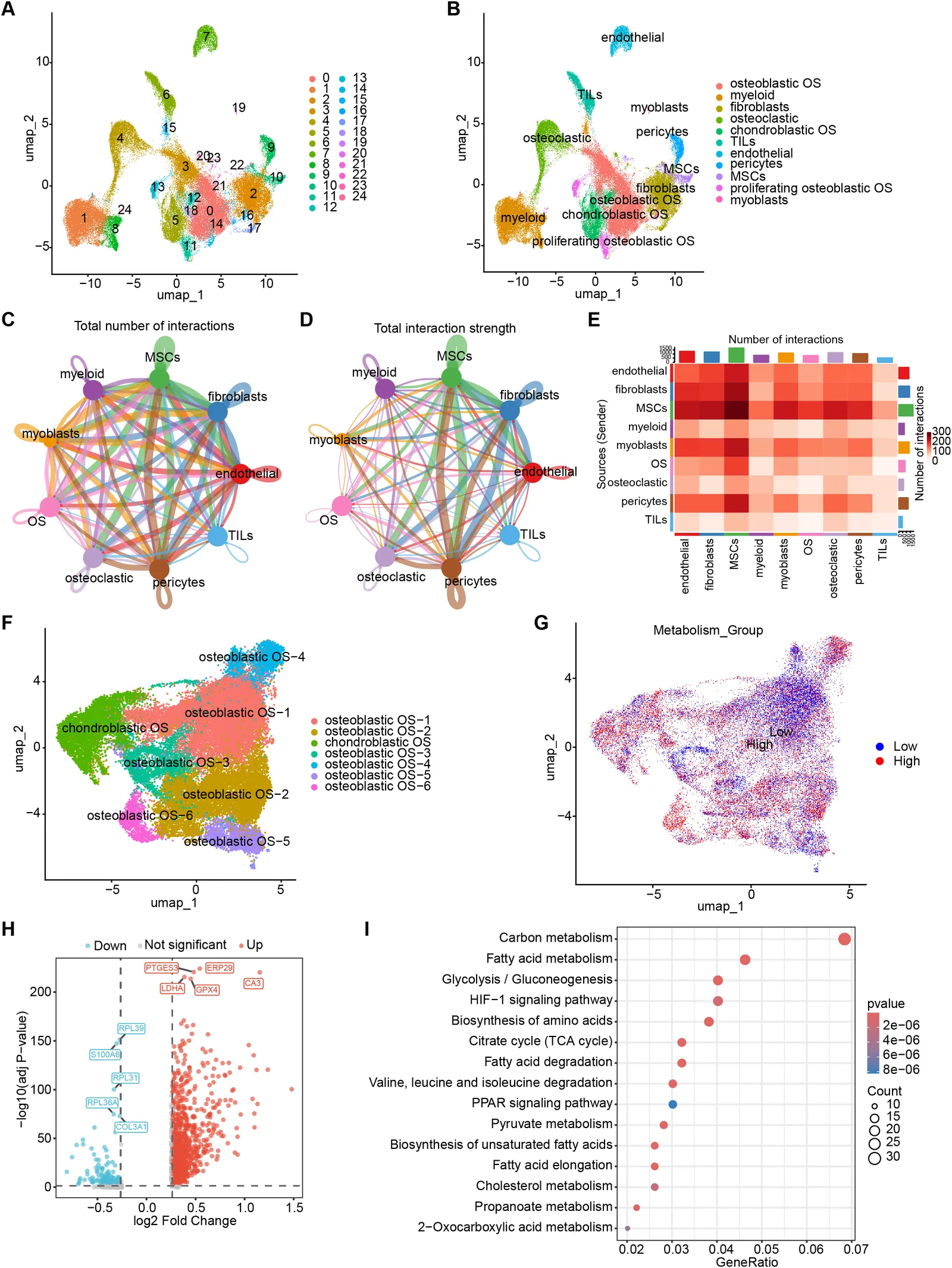
Single-cell profiling reveals lipid-metabolic heterogeneity and intercellular crosstalk in osteosarcoma. **(A)** UMAP plots showing 25 distinct cell clusters. **(B)** UMAP visualization of 11 annotated cell types in osteosarcoma samples. **(C, D)** CellChat-inferred intercellular communication networks showing the total number **(C)** and overall strength **(D)** of interactions among major cell types. **(E)** Heatmap depicting the number of inferred cell–cell interactions, with rows representing sender populations and columns representing receiver populations. **(F)** UMAP visualization of seven malignant OS cell subclusters, comprising six osteoblastic and one chondroblastic population. **(G)** UMAP visualization of OS cells stratified into high- and low-lipid-metabolism groups based on AUCell scores. **(H)** Volcano plot of DEGs between high- and low-lipid-metabolism OS cells; upregulated genes are shown in red, downregulated genes in blue. **(I)** KEGG pathway enrichment of DEGs between high- and low-lipid-metabolism OS cells.

To characterize the cellular architecture of osteosarcoma at single-cell resolution and provide a basis for subsequent lipid metabolism-related analyses, we analyzed the scRNA-seq dataset GSE152048. After stringent filtering and quality control, 101,747 high-quality cells derived from 11 osteosarcoma samples were retained for downstream analysis. Unsupervised clustering combined with UMAP visualization resolved these cells into 25 transcriptionally distinct clusters (**Figure 1A**), which were further assigned to 11 major cellular lineages based on canonical marker gene expression, including osteoblastic OS cells, myeloid cells, fibroblasts, osteoclastic cells, chondroblastic OS cells, tumor-infiltrating lymphocytes (TILs), endothelial cells, pericytes, mesenchymal stem cells (MSCs), proliferating osteoblastic OS cells, and myoblasts (**Figure 1B**). Bubble plots summarized the average expression levels and proportions of representative marker genes across defined cell types (**Supplementary Figure 1C**). In addition, the relative abundance of each cell type varied substantially among individual samples, highlighting the marked compositional diversity of the osteosarcoma tumor microenvironment (**Supplementary Figure 1B**). We next investigated potential intercellular communication within the osteosarcoma ecosystem. CellChat analysis revealed an extensive communication network connecting malignant, immune, and stromal compartments, as indicated by both the number and overall strength of inferred interactions (**Figures 1C, D**). Heatmap analysis further highlighted prominent signaling activity involving MSCs, fibroblasts, endothelial cells, and pericytes (**Figure 1E**). Notably, osteosarcoma cells showed prominent bidirectional crosstalk with these populations (**Supplementary Figures 1C, D**). To evaluate lipid metabolism activity at single-cell resolution, AUCell was used to calculate AUC scores for each cell based on a lipid metabolism-related gene set. UMAP visualization revealed heterogeneous lipid metabolic activity across cell types, with higher AUC scores mainly enriched in myeloid and osteoclastic populations (**Supplementary Figures 1E, F**). InferCNV analysis further supported the malignant identity of the annotated OS populations, which exhibited more pronounced chromosome-scale CNV alterations and significantly higher CNV scores than the TIL and myeloid reference cells (**Supplementary Figures 2A, B**). To further characterize malignant cell heterogeneity, osteosarcoma cells were extracted and reclustered. Based on marker gene expression, seven osteosarcoma subclusters were identified, including six osteoblastic osteosarcoma subpopulations and one chondroblastic osteosarcoma subpopulation (**Figure 1F**; **Supplementary Figures 2C–E**). Lipid metabolism AUC scores were then projected onto the reclustered malignant cell compartment, revealing distinct metabolic patterns across osteosarcoma subclusters and enabling stratification of osteosarcoma cells into lipid-metabolism-high and -low groups (**Figure 1G**; **Supplementary Figure 2F**). Differential expression analysis delineated a distinct transcriptional signature associated with lipid metabolic status, yielding 864 DEGs, including 717 upregulated and 147 downregulated genes (**Figure 1H**; **Supplementary Figure 2G**). GO enrichment analysis showed that these DEGs were mainly associated with fatty acid metabolic processes, lipid catabolic processes, mitochondrial matrix localization, and oxidoreductase-related functions (**Supplementary Figure 2H**). Consistently, KEGG enrichment analysis further revealed significant enrichment in carbon metabolism, fatty acid metabolism, glycolysis/gluconeogenesis, fatty acid degradation, pyruvate metabolism, PPAR signaling, and cholesterol metabolism (**Figure 1I**). Collectively, these findings reveal a heterogeneous and highly interactive osteosarcoma ecosystem in which malignant cells display distinct lipid-metabolic states linked to broad metabolic reprogramming.

### Cell trajectory analysis and intercellular communication differences in malignant OS cells with high and low lipid metabolism scores

**Figure 2:**
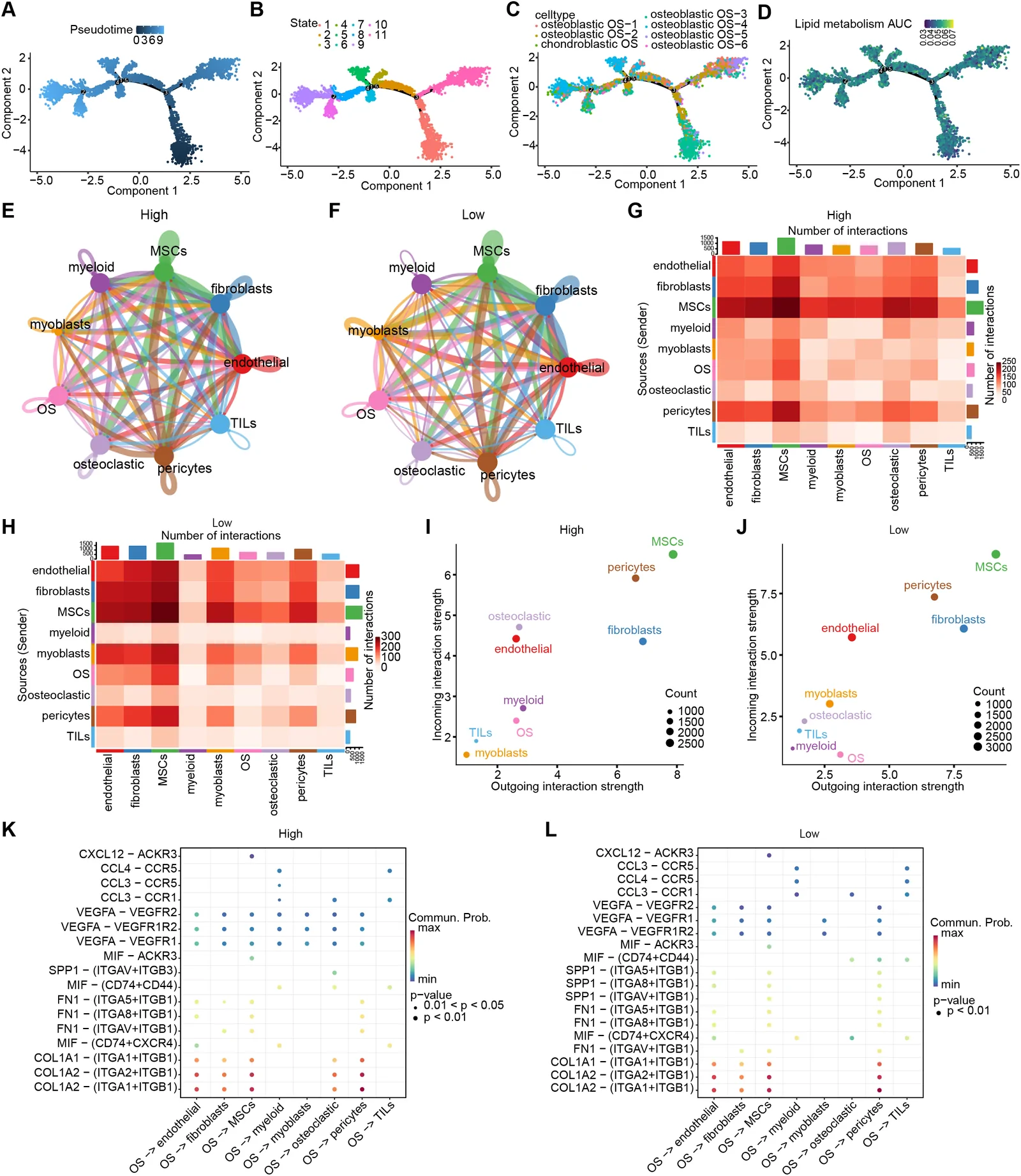
Cell trajectory analysis and intercellular communication differences in malignant OS cells with high and low lipid metabolism scores. **(A)** Monocle-based pseudotime trajectory of OS cells. **(B)** Distribution of cells across 11 pseudotemporal states. **(C)** Mapping of seven OS subclusters onto the trajectory. **(D)** Trajectory plot showing lipid metabolism AUCell scores of malignant OS cells. **(E, F)** CellChat-inferred communication networks among major cell types in the high- **(E)** and low-lipid-metabolism **(F)** groups. **(G, H)** Heatmaps of CellChat-inferred interaction numbers in the high- **(G)** and low-lipid-metabolism **(H)** groups. **(I, J)** Incoming and outgoing interaction strengths across cell populations in the high- **(I)** and low-lipid-metabolism **(J)** groups; point size indicates interaction count. **(K, L)** Bubble plots of OS-derived ligand-receptor interactions with other cell populations in the high- **(K)** and low-lipid-metabolism **(L)** groups; color indicates communication probability, and dot size reflects statistical significance.

To explore dynamic transcriptional transitions among malignant osteosarcoma subclusters, pseudotime trajectory analysis was performed using Monocle. Reclustering-derived OS cells were ordered along a branched trajectory with gradual pseudotime progression and were further partitioned into 11 cellular states (**Figures 2A, B**). As shown in **Figure 2C**, osteoblastic OS-3 was mainly distributed along the early-to-intermediate regions, whereas osteoblastic OS-1 and OS-2 spanned broader intermediate-to-late branches. In contrast, chondroblastic OS, osteoblastic OS-4, OS-5 and OS-6 were relatively enriched at later pseudotime stages. To further characterize lineage-related transcriptional dynamics, representative osteogenic and chondrogenic markers were examined along pseudotime. Osteogenic markers, including COL1A1, COL3A1, and RUNX2, showed dynamic expression changes across the trajectory, with relatively higher expression at intermediate pseudotime followed by a decline toward later stages (**Supplementary Figures 3A, B**). Conversely, chondrogenic markers such as ACAN, COL2A1, and SOX9 exhibited stage-dependent expression patterns, with COL2A1 and SOX9 tending to increase toward late pseudotime (**Supplementary Figures 3A, B**). By projecting lipid metabolism AUC scores onto the pseudotime trajectory, we found that lipid metabolic activity was dynamically remodeled during the inferred state transition of malignant OS cells (**Figure 2D**). Subcluster-specific trend analyses further showed that chondroblastic OS cells and several osteoblastic OS subpopulations, particularly osteoblastic OS-2, OS-3, OS-5, and OS-6, exhibited increased lipid metabolism scores toward intermediate-to-late pseudotime stages, whereas osteoblastic OS-1 and OS-4 showed relatively modest fluctuations across the trajectory (**Supplementary Figure 3C**). These results indicate that lipid metabolic activity is dynamically remodeled along the inferred malignant osteosarcoma differentiation trajectory.

Given these dynamic metabolic differences, we next determined whether lipid metabolic status was associated with altered intercellular communication. CellChat analysis was performed separately in the high- and low-lipid-metabolism osteosarcoma groups. Both groups exhibited extensive communication networks among malignant, stromal, and immune cell populations, although the overall interaction patterns differed between the two metabolic states (**Figures 2E, F**). Heatmap and interaction-strength analyses showed that MSCs, fibroblasts, endothelial cells, and pericytes contributed prominently to the inferred signaling network in both groups (**Figures 2G–J**). Focusing on osteosarcoma-centered communication, we observed that osteosarcoma cells maintained bidirectional interactions with multiple microenvironmental populations in both lipid-metabolism groups, whereas these interactions were more pronounced in the high-lipid-metabolism group (**Supplementary Figures 4A–D**). Ligand-receptor analysis further showed that osteosarcoma-derived communication in both lipid-metabolism groups was predominantly mediated by collagen-integrin signaling, while chemokine-, VEGF-, MIF-, FN1-, and SPP1-related interactions were also detected between osteosarcoma cells and surrounding microenvironmental populations (**Figures 2K, L**). Similar signaling modules were also observed when osteosarcoma cells acted as receivers, suggesting reciprocal communication between malignant cells and the surrounding microenvironment (**Supplementary Figures 4E, F**). Collectively, these findings indicate that lipid metabolic status is closely associated with the remodeling of osteosarcoma-centered intercellular communication, particularly through stromal, extracellular matrix, angiogenic, and chemokine-related signaling programs.

### WGCNA and single-cell integration identify LMRGs in osteosarcoma

**Figure 3:**
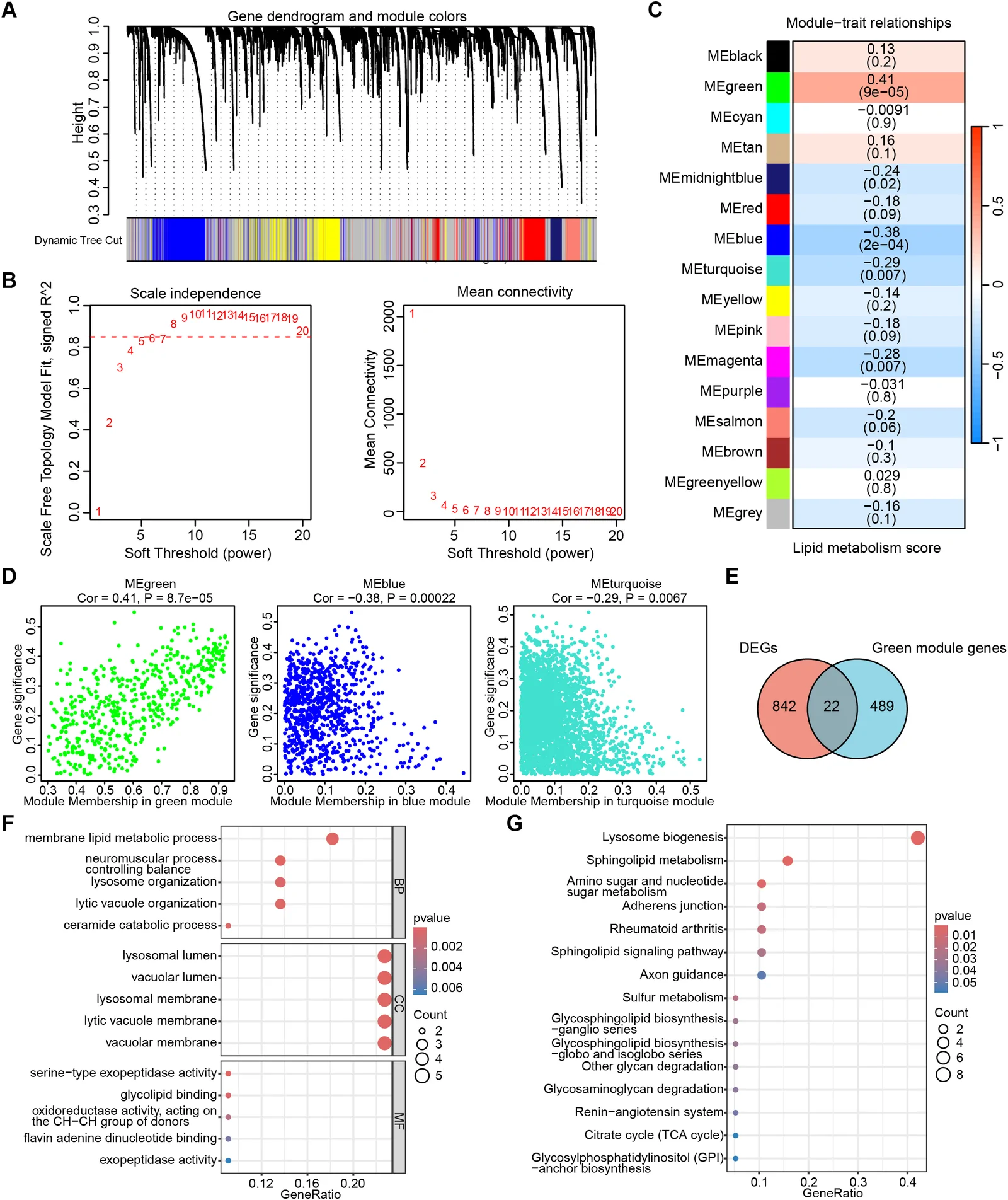
WGCNA and single-cell integration identify LMRGs in osteosarcoma. **(A)** WGCNA gene dendrogram with color-coded co-expression modules. **(B)** Selection of the WGCNA soft-thresholding power based on scale-free topology fit and mean connectivity; β = 8 was selected, corresponding to a signed R² of 0.911. **(C)** Module-trait relationship heatmap showing correlations between WGCNA modules and lipid metabolism scores, with the green module exhibiting the strongest positive association. **(D)** Scatter plots showing the relationships between gene significance and module membership in the green, blue, and turquoise modules. **(E)** Venn diagram showing 22 genes shared between DEGs from high- and low-lipid-metabolism OS cells and the WGCNA green module. **(F, G)** GO **(F)** and KEGG **(G)** enrichment analyses of the 22 overlapping genes.

To identify bulk transcriptome-derived co-expression modules associated with lipid metabolism, WGCNA was performed using the TARGET-OS cohort. After constructing a scale-free co-expression network, an optimal soft-thresholding power of β = 8 was selected, at which the scale-free topology fit reached a signed R² value of 0.911, indicating that the network satisfied the scale-free distribution criterion. Hierarchical clustering subsequently classified genes into 16 color-coded modules, including 15 non-grey modules and the grey module (**Figures 3A, B**). Correlation analysis between module eigengenes and lipid metabolism scores revealed that the green module, containing 511 genes, showed the strongest positive association with lipid metabolism status (Cor = 0.41, *P* = 9e-05), whereas the blue and turquoise modules were negatively correlated with lipid metabolism scores (**Figures 3C, D**). To obtain robust lipid metabolism-associated candidate genes, genes from the green module were intersected with DEGs identified between high- and low-lipid-metabolism OS cells in the single-cell analysis, yielding 22 overlapping genes for subsequent investigation (**Figure 3E**). GO enrichment analysis indicated that these genes were predominantly implicated in membrane lipid metabolic processes, glycolipid binding, and oxidoreductase activity (**Figure 3F**). Consistently, KEGG pathway analysis showed significant enrichment in sphingolipid metabolism, glycosphingolipid biosynthesis, and sphingolipid signaling pathways (**Figure 3G**). Together, these results integrate bulk and single-cell transcriptomic evidence to define a focused set of LMRGs that may contribute to osteosarcoma metabolic heterogeneity.

### Construction of a LMRLs signature for osteosarcoma prognosis in the training cohort

**Figure 4:**
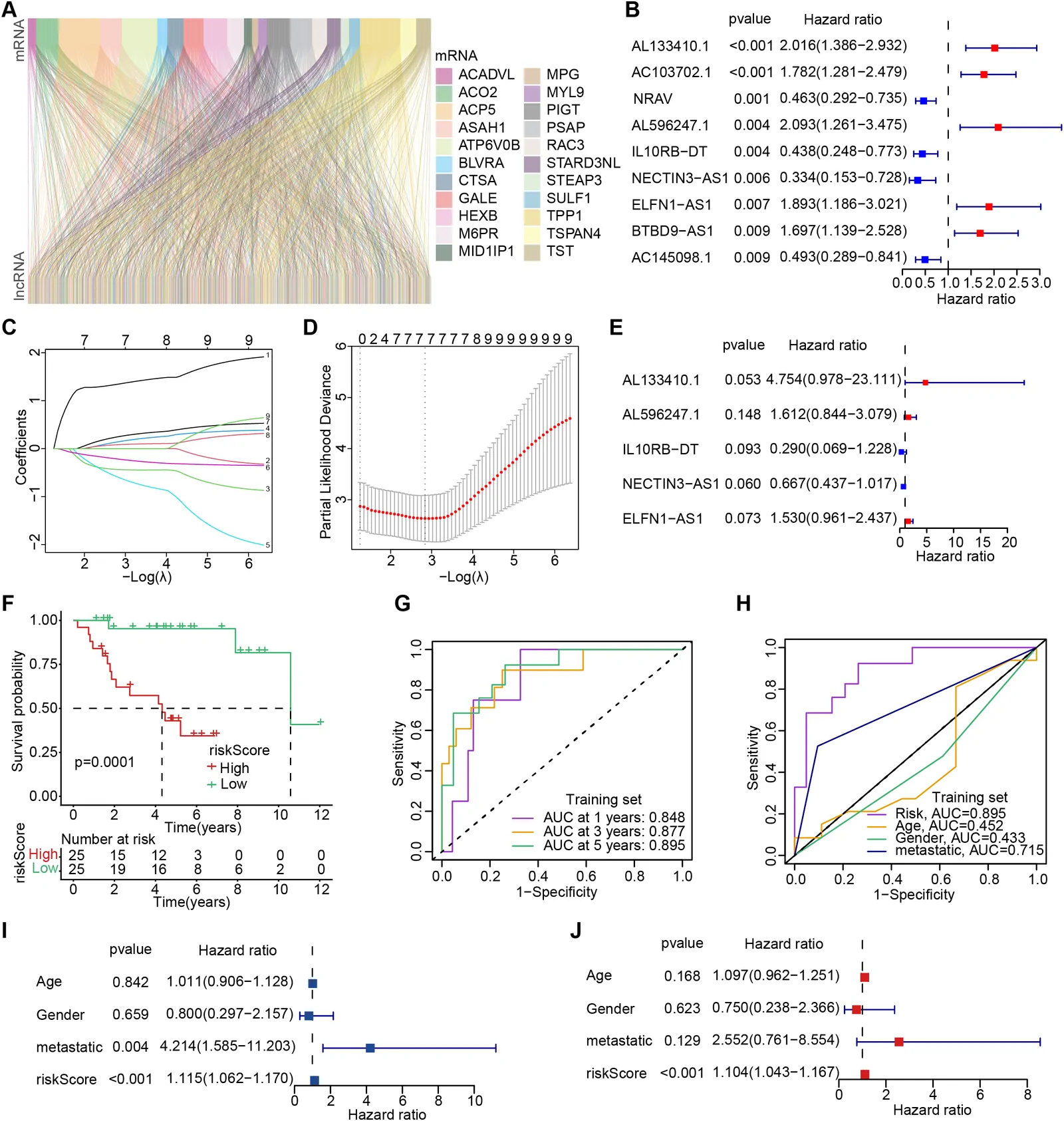
Construction of a LMRL signature for osteosarcoma prognosis in the training cohort. **(A)** Sankey diagram illustrating co-expression relationships between the 22 overlapping genes and their correlated lncRNAs in osteosarcoma. **(B)** Univariate Cox regression identified nine prognostic LMRLs. **(C, D)** LASSO Cox regression showing coefficient trajectories and optimal λ selection. **(E)** Five LMRLs retained by multivariable Cox analysis were used to establish the prognostic signature. **(F)** Kaplan-Meier analysis showing significantly poorer overall survival in the high-risk group within the training cohort (*P* = 0.0001). **(G)** Time-dependent ROC curves of the prognostic signature for predicting 1-, 3-, and 5-year overall survival in the training cohort (AUCs = 0.848, 0.877, and 0.895, respectively). **(H)** ROC curves comparing the prognostic performance of the risk score and clinical variables in the training cohort (AUCs: risk score, 0.895; age, 0.452; gender, 0.433; metastatic status, 0.715). **(I, J)** Univariate **(I)** and multivariable **(J)** Cox regression analyses of the risk score and clinical variables in the training cohort.

We next sought to develop a lncRNA-based prognostic signature linked to these metabolic programs. Pearson co-expression analysis was performed between the 22 candidate genes and lncRNAs using |cor| > 0.30 and *P* < 0.001 as filtering criteria, through which 958 co-expressed LMRLs were identified and visualized using a Sankey diagram (**Figure 4A**). Subsequently, univariate Cox regression analysis identified nine LMRLs that were significantly associated with overall survival in the training cohort (**Figure 4B**). To further optimize candidate selection, LASSO Cox regression was performed using the optimal penalty parameter λ, after which the retained LMRLs were incorporated into multivariate Cox regression analysis (**Figures 4C–E**). Ultimately, a five-LMRL prognostic signature was established, with the risk score calculated as follows: Risk score = (AL133410.1 expression) * 1.559 + (AL596247.1 expression) * 0.478 + (ELFN1-AS1 expression) * 0.426 + (IL10RB-DT expression) * -1.237 + (NECTIN3-AS1 expression) * -0.405. Based on the median risk score derived from this signature, patients in the training cohort were further stratified into high-risk and low-risk groups. The risk distribution and survival status plots showed that increasing risk scores were accompanied by shortened survival time and a higher frequency of death events (**Supplementary Figures 5A, B**). Consistently, the expression heatmap revealed distinct expression patterns of the five signature lncRNAs between the two risk groups (**Supplementary Figure 5C**). Furthermore, Kaplan-Meier survival analysis demonstrated that patients in the high-risk group had significantly poorer overall survival than those in the low-risk group (p = 0.0001; **Figure 4F**). Time-dependent ROC analysis further indicated favorable predictive performance of the signature, with AUC values of 0.848, 0.877, and 0.895 for 1-, 3-, and 5-year overall survival, respectively (**Figure 4G**). Moreover, when compared with conventional clinicopathological variables, the risk score showed superior prognostic discrimination, exceeding age, gender, and metastatic status in predictive accuracy (**Figure 4H**). Finally, univariate Cox regression analysis identified both metastatic status and the risk score as significant prognostic factors, while multivariate Cox regression confirmed that the risk score remained an independent predictor of overall survival after adjustment for age, gender, and metastatic status (**Figures 4I, J**). Collectively, these findings demonstrate that the five-LMRL signature provides a robust and independently informative tool for survival stratification in osteosarcoma patients in the training cohort.

### Validation and nomogram-based clinical translation of the LMRLs risk model

**Figure 5:**
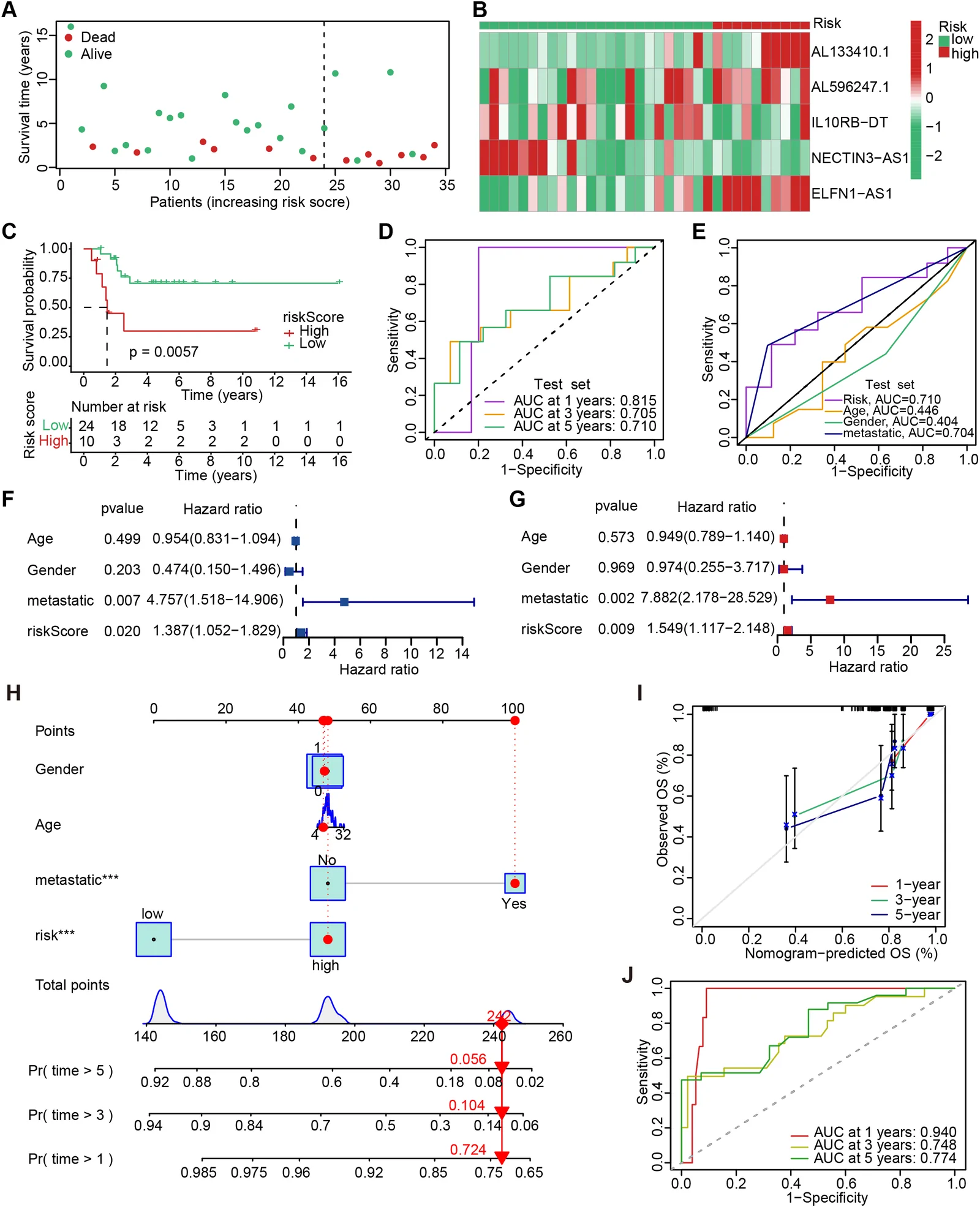
Validation and nomogram-based clinical translation of the LMRL risk model. **(A, B)** Survival status and five-LMRL expression profiles in the test group. **(C)** Kaplan-Meier analysis showing significantly poorer overall survival in the high-risk group within the test group (*P* = 0.0057). **(D)** Time-dependent ROC curves of the prognostic signature for predicting 1-, 3-, and 5-year overall survival in the test group (AUCs = 0.815, 0.705, and 0.710, respectively). **(E)** ROC curves comparing the prognostic performance of the risk score and clinical variables in the test group (AUCs: risk score, 0.710; age, 0.446; gender, 0.404; metastatic status, 0.704). **(F, G)** Univariate **(F)** and multivariable **(G)** Cox regression analyses of the risk score and clinical variables in the test group. **(H)** Clinical nomogram for individualized prediction of 1-, 3-, and 5-year overall survival in osteosarcoma. **(I)** Calibration curves assessing agreement between predicted and observed 1-, 3-, and 5-year overall survival. **(J)** Time-dependent ROC curves evaluating the nomogram for 1-, 3-, and 5-year overall survival prediction (AUCs = 0.940, 0.748, and 0.774, respectively).

To evaluate the robustness of the LMRLs risk model, we further validated its prognostic performance in the testing cohort. As the risk score increased, death events became more frequent, while the five signature LMRLs showed distinct expression patterns between the high- and low-risk groups (**Figures 5A, B**). Kaplan-Meier analysis confirmed that high-risk patients had significantly worse overall survival than low-risk patients (p = 0.0057; **Figure 5C**). Time-dependent ROC analysis showed that the model retained favorable predictive performance, with AUC values of 0.815, 0.705, and 0.710 for predicting 1-, 3-, and 5-year overall survival, respectively (**Figure 5D**). Moreover, the risk score showed better prognostic discrimination than age, gender, and metastatic status, and Cox regression analyses confirmed that both metastatic status and risk score were independent prognostic factors in the testing cohort (**Figures 5E–G**). We next assessed the model in the combined cohort to further examine its overall stability. Consistent with the testing cohort, high-risk patients showed increased mortality, distinct signature-LMRL expression patterns, and significantly poorer overall survival (p = 0.0003; **Supplementary Figures 6A–C**). Time-dependent ROC analysis yielded AUC values of 0.833, 0.804, and 0.809 for 1-, 3-, and 5-year overall survival prediction, respectively (**Supplementary Figure 6D**). In addition, the risk score outperformed conventional clinical variables and remained an independent prognostic factor together with metastatic status (**Supplementary Figures 6E–G**).

To facilitate individualized survival prediction for osteosarcoma patients, we further constructed a nomogram by integrating the LMRLs risk score with clinical variables, including age, gender, and metastatic status. In this nomogram, each variable was assigned a corresponding point value, and the cumulative total score was used to estimate 1-, 3-, and 5-year overall survival probabilities. Notably, the risk score and metastatic status contributed prominently to the total points, suggesting their major prognostic weight in survival prediction (**Figure 5H**). Calibration curve analysis showed acceptable agreement between the nomogram-predicted and observed overall survival probabilities at 1, 3, and 5 years, indicating favorable model calibration (**Figure 5I**). Furthermore, time-dependent ROC analysis demonstrated that the nomogram achieved AUC values of 0.940, 0.748, and 0.774 for predicting 1-, 3-, and 5-year overall survival, respectively (**Figure 5J**). Collectively, these results demonstrate that the LMRLs risk model exhibits stable prognostic performance and that its nomogram-based integration with clinical characteristics may provide a practical tool for individualized survival assessment in osteosarcoma.

### Functional, immune, genomic, and therapeutic heterogeneity between LMRLs risk subgroups

Differential expression analysis was performed to explore the biological differences between the high- and low-risk groups, identifying 679 DEGs, including 330 upregulated and 349 downregulated genes in the high-risk group (**Supplementary Figures 7A, B**). GO enrichment analysis showed that these DEGs were mainly involved in extracellular matrix organization, glycosaminoglycan binding, cytokine activity, and growth factor binding (**Supplementary Figure 7C**). KEGG analysis further revealed enrichment in PI3K-Akt signaling, MAPK signaling, Wnt signaling, ECM-receptor interaction, and Rap1 signaling pathways, suggesting that risk stratification was associated with extracellular matrix remodeling and tumor progression-related signaling programs (**Supplementary Figure 7D**).

**Figure 6:**
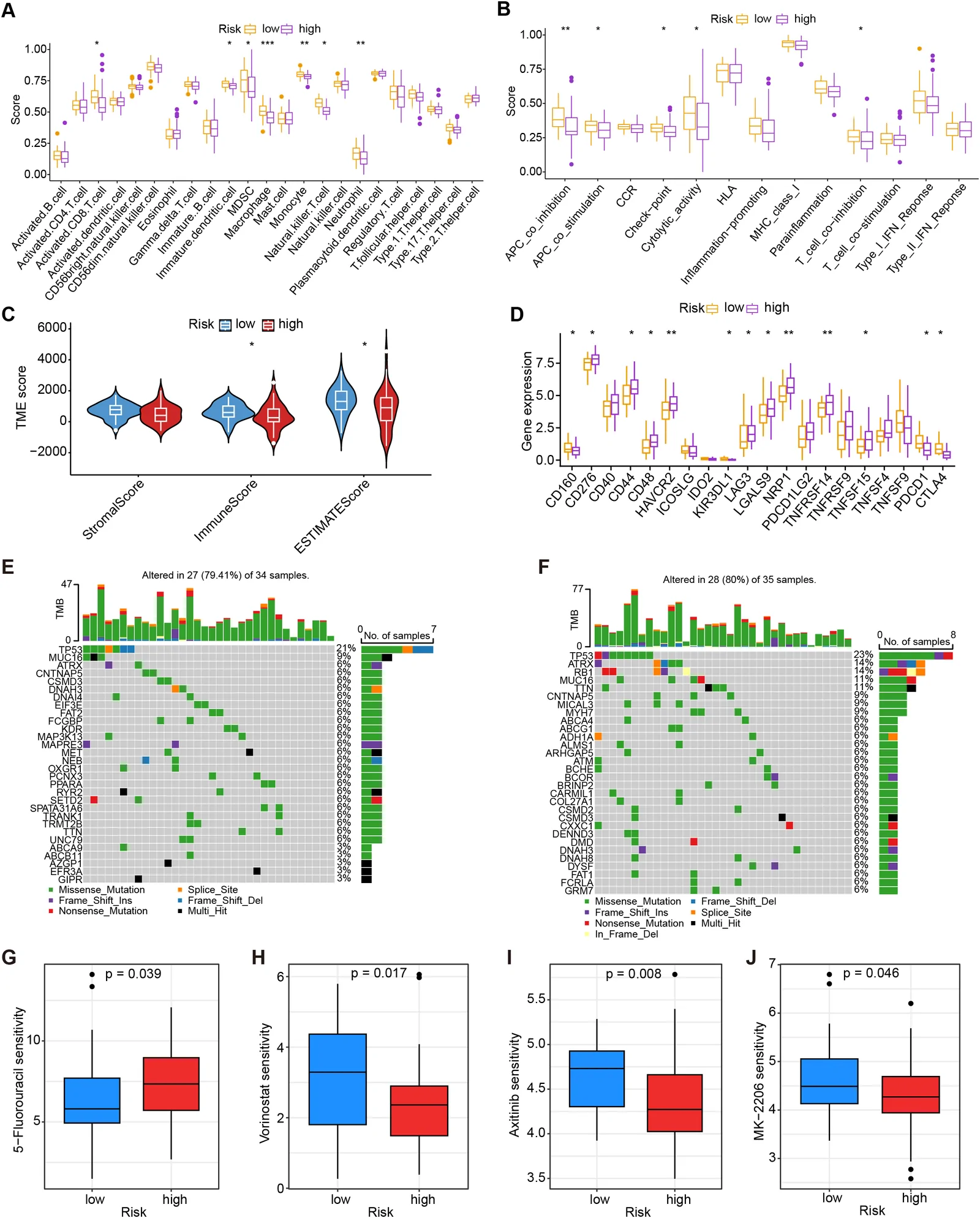
Functional, immune, genomic, and therapeutic heterogeneity between LMRL risk subgroups. **(A)** Comparison of ssGSEA-derived immune cell infiltration scores between the low- and high-risk osteosarcoma groups. **(B)** Boxplots showing ssGSEA-estimated immune functional activity in the low- and high-risk osteosarcoma groups. **(C)** Comparison of StromalScore, ImmuneScore, and ESTIMATEScore between the low- and high-risk groups. **(D)** Comparison of immune checkpoint gene expression between the low- and high-risk osteosarcoma groups. **(E, F)** Waterfall plots depicting the somatic mutation landscapes of the low-risk **(E)** and high-risk **(F)** osteosarcoma groups. **(G–J)** Comparison of predicted sensitivity to 5-fluorouracil **(G)**, vorinostat **(H)**, axitinib **(I)**, and MK-2206 **(J)** between the low- and high-risk groups. *P < 0.05, **P < 0.01, ***P < 0.001.

Given the enrichment of cytokine-related and tumor microenvironment-associated pathways, we further explored immune-related features between the high- and low-risk groups via exploratory computational analysis. Given that the enrichment of cytokine-related and tumor microenvironment-associated pathways, we further characterized the immune landscape between the high- and low-risk groups. ssGSEA analysis revealed distinct immune-cell infiltration patterns between the high- and low-risk groups, among which natural killer T cells, activated CD8 T cells, macrophages, and neutrophils showed significantly higher infiltration scores in the low-risk group (**Figure 6A**). In parallel, immune functional profiling showed that several immune-related signatures, including APC co-inhibition, APC co-stimulation, checkpoint activity, cytolytic activity, and T-cell co-inhibition, were significantly elevated in the low-risk group (**Figure 6B**). ESTIMATE analysis further demonstrated that the low-risk group had higher immune and ESTIMATE scores, whereas the high-risk group exhibited increased tumor purity, hinting a relatively less immune-infiltrated microenvironment in high-risk patients (**Figure 6C**; **Supplementary Figure 8A**). Moreover, several immune checkpoint-related genes differed significantly between the two groups, among which CD276, CD44, CD48, HAVCR2, LAG3, LGALS9, NRP1, TNFRSF14, and TNFSF15 were upregulated in the high-risk group, whereas CD160, PDCD1, and CTLA4 were higher in the low-risk group (**Figure 6D**).

Somatic mutation profiling showed comparable overall mutation frequencies between the low- and high-risk groups, with mutations detected in 27 of 34 low-risk samples (79.41%) and 28 of 35 high-risk samples (80.00%) (**Figures 6E, F**). Missense mutations represented the predominant variant type in both groups, while TP53 was the most frequently mutated gene. In the low-risk group, MUC16, ATRX, CNTNAP5, CSMD3, and DNAH3 were among the recurrently mutated genes, whereas ATRX, RB1, MUC16, TTN, CNTNAP5, MICAL3, and MYH7 showed relatively higher mutation frequencies in the high-risk group (**Figures 6E, F**). In addition, the high-risk group exhibited significantly higher TMB than the low-risk group (*P* = 0.01; **Supplementary Figure 8B**), and risk score was weakly but significantly correlated with TMB (R = 0.27, *P* = 0.023; **Supplementary Figure 8C**), suggesting that higher risk scores were accompanied by increased mutational burden. Drug sensitivity prediction further indicated risk-associated therapeutic heterogeneity. Compared with the low-risk group, the high-risk group showed significantly lower predicted IC_50_ values for several clinically relevant or targeted agents, including vorinostat, axitinib, sorafenib, linsitinib, MK-2206, and LY2109761, suggesting that high-risk patients may be more sensitive to these compounds (**Figures 6H–J**; **Supplementary Figure 8D**). By contrast, the low-risk group exhibited lower predicted IC_50_ values for 5-fluorouracil, trametinib, and ruxolitinib, indicating subgroup-specific drug response patterns (**Figure 6G**; **Supplementary Figure 8D**). Collectively, these exploratory in silico observations suggest that the LMRL risk signature correlates with divergent biological programs, immune microenvironmental profiles, genomic alteration features and predicted therapeutic vulnerabilities in osteosarcoma. All associations derived from computational inference require further experimental and clinical validation.

### ELFN1-AS1 is prioritized as a key LMRL in osteosarcoma

Kaplan-Meier analysis further confirmed the distinct prognostic relevance of the five signature LMRLs. High expression of AL133410.1, AL596247.1, and ELFN1-AS1 was associated with poorer overall survival, whereas elevated IL10RB-DT and NECTIN3-AS1 indicated favorable survival outcomes (**Supplementary Figures 9A–E**). Time-dependent ROC analysis showed that ELFN1-AS1 and AL133410.1 exhibited relatively stronger individual predictive value, with 1-, 3-, and 5-year AUCs of 0.763, 0.684, and 0.683 for ELFN1-AS1, and 0.661, 0.727, and 0.793 for AL133410.1, respectively (**Supplementary Figures 9F–J**). Consistent with these bioinformatic findings, ELFN1-AS1 was the most markedly upregulated candidate LMRL in osteosarcoma tissues relative to matched adjacent normal controls, providing a rationale for its further functional and mechanistic investigation.

**Figure 7:**
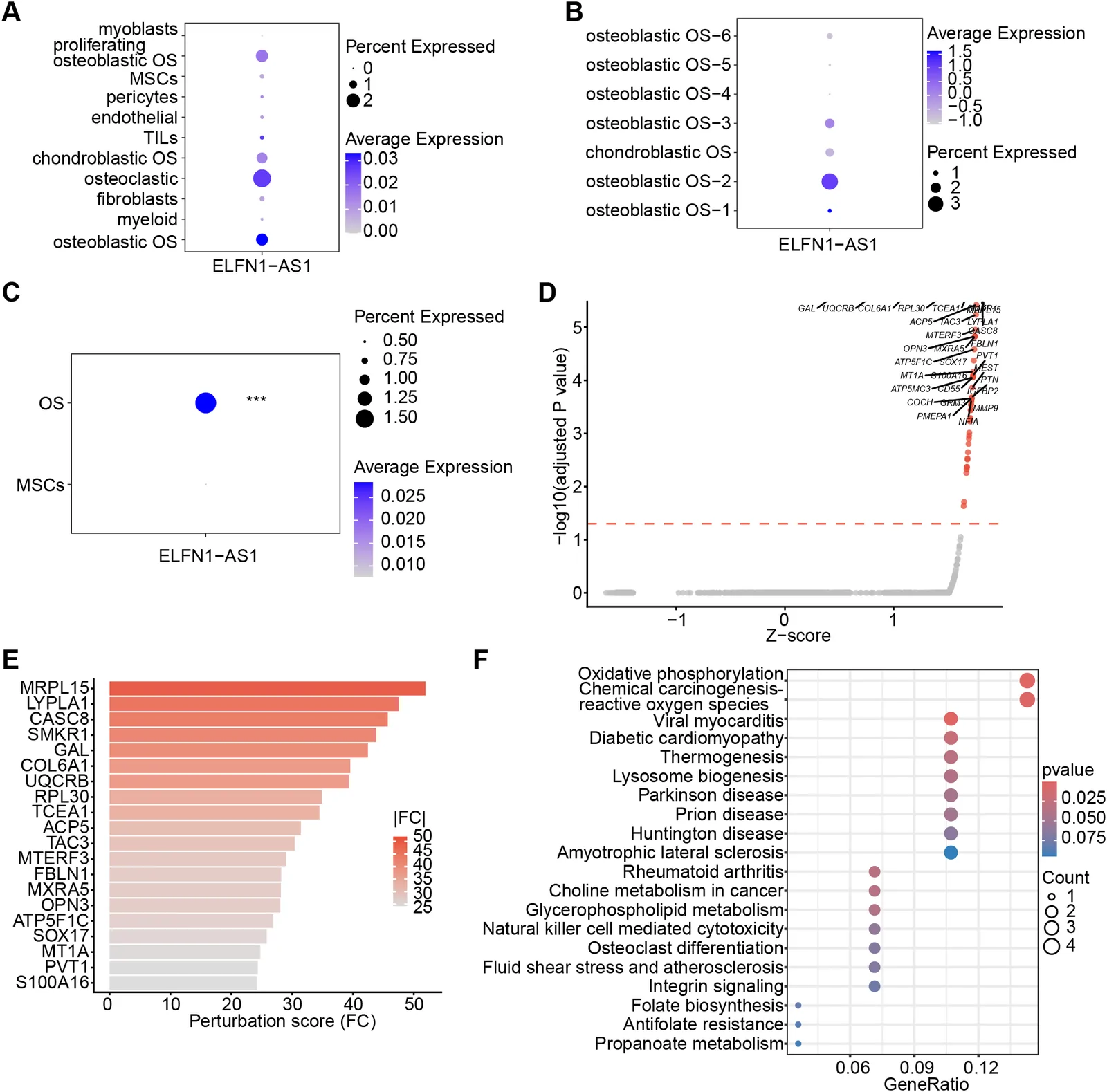
ELFN1-AS1 is prioritized as a key LMRL in osteosarcoma. **(A)** Dot plot showing ELFN1-AS1 expression across annotated cell types. **(B)** Dot plot showing ELFN1-AS1 expression across seven OS cell subclusters. **(C)** Dot plot comparing ELFN1-AS1 expression between OS cells and MSCs. **(D)** Volcano plot showing downstream transcriptional changes following virtual knockout of ELFN1-AS1. **(E)** Bar plot showing the top 20 genes with the highest perturbation scores following virtual knockout of ELFN1-AS1. **(F)** KEGG pathway enrichment analysis of significantly altered genes following virtual knockout of ELFN1-AS1.

Single-cell expression analysis further revealed a cell-type-specific distribution of ELFN1-AS1 within the osteosarcoma microenvironment. ELFN1-AS1 was preferentially expressed in malignant OS cells compared with most non-malignant cell populations (**Figure 7A**; **Supplementary Figure 10A**), with particularly enriched expression in the osteoblastic OS-2 subpopulation (**Figure 7B**; **Supplementary Figure 10B**). Moreover, comparison between OS cells and MSCs further revealed significantly higher ELFN1-AS1 expression in the malignant OS compartment (**Figure 7C**). To further infer the potential regulatory effects of ELFN1-AS1 at the single-cell level, we performed an in silico knockout analysis using scTenifoldKnk. Virtual perturbation of ELFN1-AS1 induced significant transcriptomic changes in a subset of downstream genes, among which MRPL15, LYPLA1, CASC8, SMKR1, GAL, COL6A1, and UQCRB showed the strongest perturbation scores (**Figures 7D, E**). KEGG enrichment analysis further showed that the perturbed genes were mainly associated with oxidative phosphorylation, reactive oxygen species-related pathways, glycerophospholipid metabolism, and choline metabolism in cancer (**Figure 7F**). Collectively, these results demonstrate that ELFN1-AS1 is preferentially upregulated in malignant osteosarcoma cells and may serve as a promising biomarker for tumor progression and unfavorable clinical outcome.

### ELFN1-AS1 is highly expressed and correlates with poor prognosis in osteosarcoma

**Figure 8:**
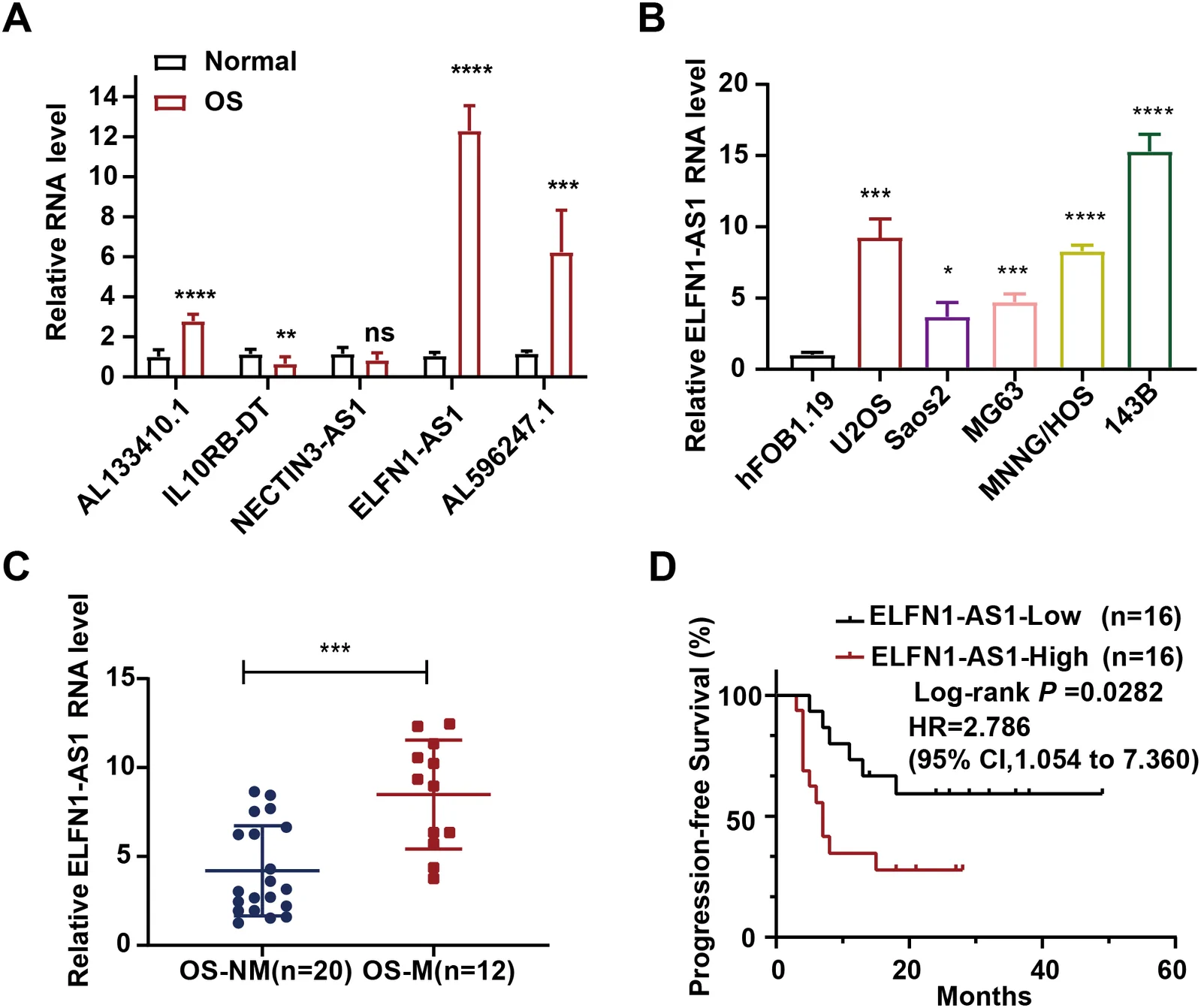
ELFN1-AS1 is highly expressed and correlates with poor prognosis in osteosarcoma. **(A)** Relative expression levels of five LMRLs in 32 pairs of osteosarcoma tissues and matched adjacent normal bone tissues detected by RT-qPCR. **(B)** ELFN1-AS1 expression in normal osteoblast cell line hFOB1.19 and osteosarcoma cell lines (U2OS, Saos2, MG63, MNNG/HOS, 143B). **(C)** ELFN1-AS1 expression in metastatic (n=12) and non-metastatic (n=20) osteosarcoma tissues. **(D)** Kaplan-Meier progression-free survival analysis of osteosarcoma patients stratified by ELFN1-AS1 expression (log-rank test). All data are presented as mean ± SD. *P < 0.05, **P < 0.01, ***P < 0.001, ****P < 0.0001; ns, not significant.

To validate the LMRLs signature experimentally, we detected the expression of five signature LMRLs in 32 paired osteosarcoma and matched adjacent normal tissues via RT-qPCR (**Figure 8A**). ELFN1-AS1 exhibited the most significant upregulation in tumor tissues, while the other LMRLs showed mild or non-significant expression changes. *In vitro* validation further confirmed elevated ELFN1-AS1 levels in multiple osteosarcoma cell lines (U2OS, Saos2, MG63, MNNG/HOS, 143B) compared with normal hFOB1.19 osteoblasts, with the highest expression in aggressive 143B and U2OS cells (**Figure 8B**). These results indicate consistent ELFN1-AS1 overexpression in both clinical specimens and osteosarcoma cell lines. We further analyzed the clinical correlation of ELFN1-AS1 expression in our OS cohort (**Supplementary Table 2**). Stratification based on median ELFN1-AS1 expression revealed that high ELFN1-AS1 levels were significantly associated with distant metastasis and advanced Enneking stage, whereas no correlations were observed with age, gender, tumor size or primary site. Consistently, ELFN1-AS1 expression was markedly higher in metastatic than in non-metastatic osteosarcoma tissues (**Figure 8C**). Kaplan-Meier survival analysis demonstrated that patients with high ELFN1-AS1 expression had significantly shorter progression-free survival (**Figure 8D**). Collectively, ELFN1-AS1 is a clinically meaningful upregulated LMRL closely correlated with metastasis, advanced tumor stage and adverse prognosis in osteosarcoma, supporting its pivotal functional role in tumor progression.

### Functional validation of ELFN1-AS1 as an oncogenic driver of osteosarcoma progression *in vitro* and *in vivo*

**Figure 9:**
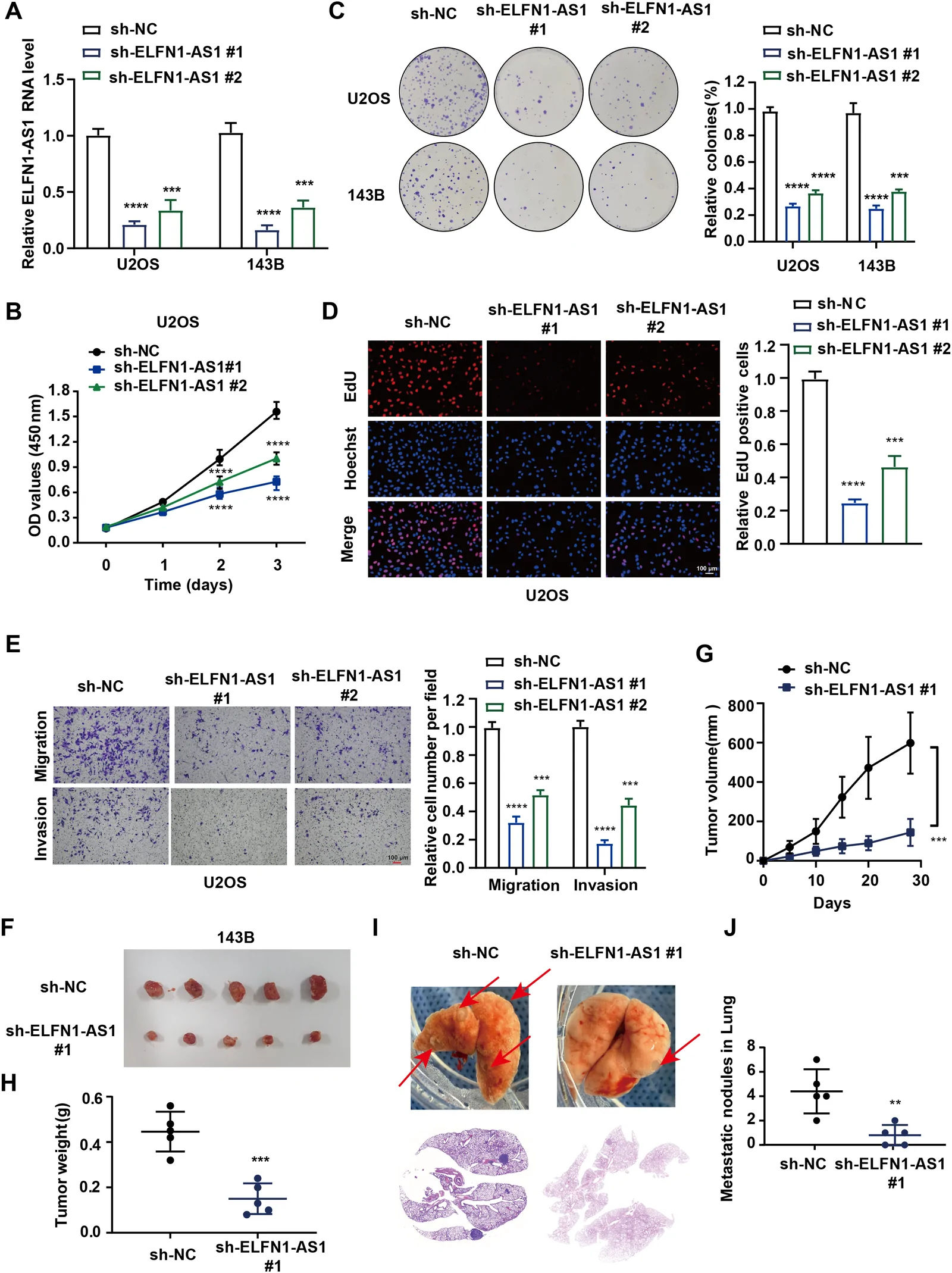
Functional validation of ELFN1-AS1 as an oncogenic driver of osteosarcoma progression *in vitro* and *in vivo*. **(A)** RT-qPCR analysis confirmed the knockdown efficiency of ELFN1-AS1 in U2OS and 143B cells transfected with sh-NC or two independent sh-ELFN1-AS1 constructs. **(B)** CCK-8 assays showed that ELFN1-AS1 knockdown significantly inhibited cell proliferation in U2OS cells. **(C)** Colony formation assays revealed reduced colony-forming ability in ELFN1-AS1-silenced U2OS and 143B cells. **(D)** EdU assays demonstrated that ELFN1-AS1 knockdown decreased the proportion of proliferative cells in U2OS cells. Scale bar: 100 μm. **(E)** Transwell migration and invasion assays showed that ELFN1-AS1 knockdown suppressed the migratory and invasive capacities of U2OS cells. Scale bar: 100 μm. **(F)** Representative images of subcutaneous xenograft tumors formed by sh-NC and sh-ELFN1-AS1#1 143B cells. **(G)** Tumor growth curves of subcutaneous xenografts measured every 5 days for 30 days. **(H)** Tumor weights at the endpoint of the xenograft experiment. **(I)** Representative images of mouse lungs with metastatic nodules (red arrows) and corresponding H&E staining. **(J)** Quantification of metastatic nodules in the lungs of mice injected with sh-NC or sh-ELFN1-AS1#1 cells. All data are presented as mean ± SD. **P < 0.01, ***P < 0.001, ****P < 0.0001.

To explore the biological functions of ELFN1-AS1 in osteosarcoma progression, loss-of-function experiments were performed in U2OS and 143B cells using two independent shRNAs. RT-qPCR confirmed effective ELFN1-AS1 knockdown, with sh-ELFN1-AS1#1 exhibiting superior silencing efficiency (**Figure 9A**). Functional assays showed that ELFN1-AS1 depletion significantly suppressed osteosarcoma cell proliferation, as evidenced by decreased cell viability in CCK-8 assays (**Figure 9B**; **Supplementary Figure 11A**), impaired colony formation capacity (**Figure 9C**), and reduced EdU-positive proliferative cells (**Figure 9D**; **Supplementary Figure 11B**). Transwell assays further verified that ELFN1-AS1 knockdown markedly inhibited the migration and invasion abilities of osteosarcoma cells (**Figure 9E**; **Supplementary Figure 11C**). A subcutaneous xenograft model was established using stably transfected 143B cells for *in vivo* validation. ELFN1-AS1 knockdown remarkably retarded tumor growth and reduced tumor volume and weight during the 28-day observation period (**Figures 9F–H**). Lung metastasis evaluation showed that the number of pulmonary metastatic nodules was significantly decreased in the sh-ELFN1-AS1#1 group (**Figures 9I, J**). Collectively, these results demonstrate that ELFN1-AS1 acts as an oncogenic lncRNA that promotes osteosarcoma proliferation, migration, invasion, tumor growth and lung metastasis, thereby facilitating malignant progression of osteosarcoma.

### ELFN1-AS1 drives osteosarcoma malignant progression via activating LYPLA1-mediated lipid accumulation-associated phenotype

**Figure 10:**
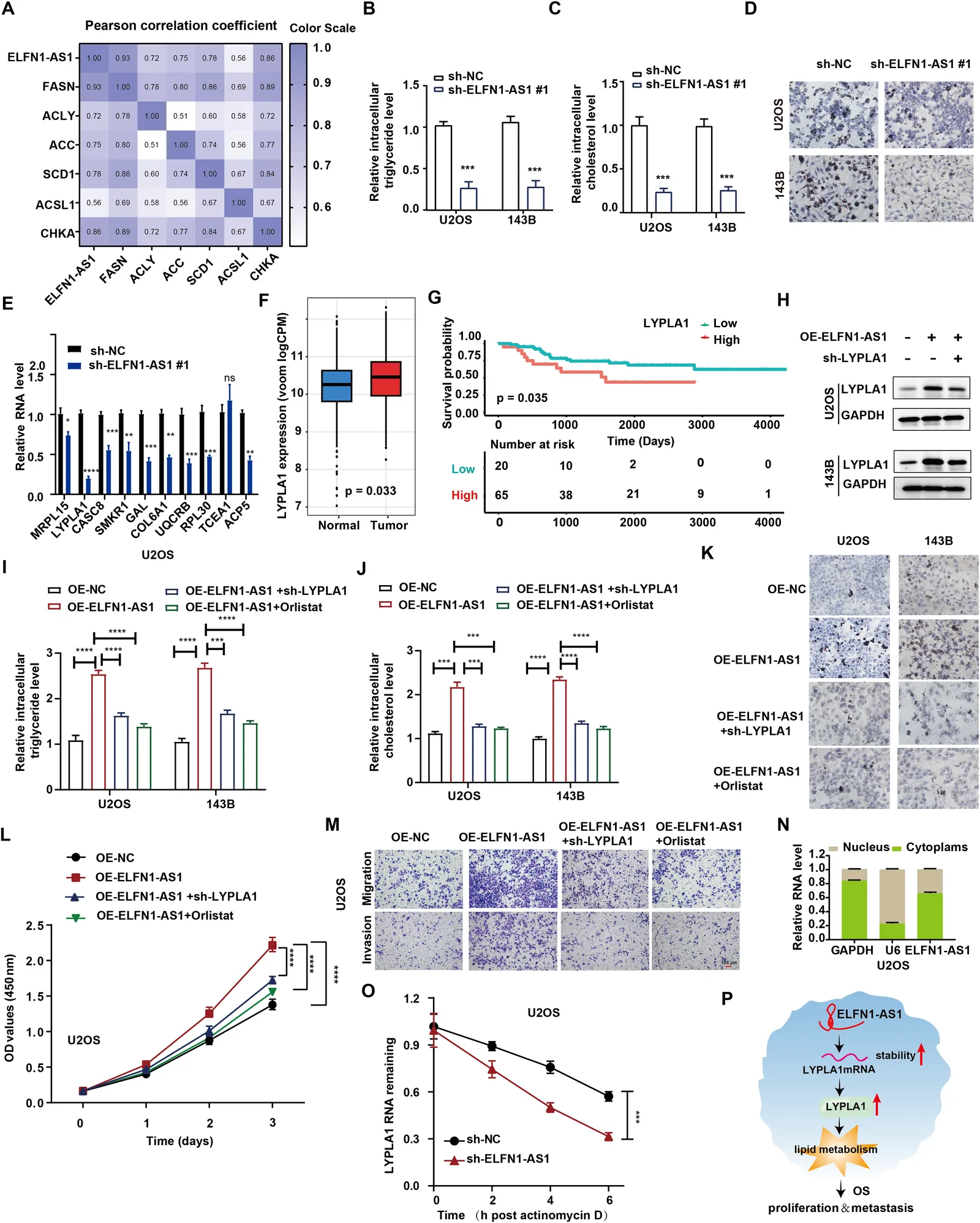
ELFN1-AS1 drives osteosarcoma malignant progression via activating LYPLA1-mediated lipid accumulation-associated phenotype. **(A)** Pearson correlation analysis between ELFN1-AS1 and key canonical lipid metabolism genes in osteosarcoma. **(B, C)** Intracellular triglyceride and cholesterol levels in sh-NC and sh-ELFN1-AS1#1 osteosarcoma cells. **(D)** Oil Red O staining in sh-NC and sh-ELFN1-AS1#1 osteosarcoma cells. **(E)** RT-qPCR analysis of candidate target genes expression in U2OS cells with ELFN1-AS1 knockdown. **(F)** LYPLA1 expression in normal and osteosarcoma tissues (TARGET data). **(G)** Kaplan-Meier survival analysis of osteosarcoma patients stratified by LYPLA1 expression. **(H)** Western blot analysis of LYPLA1 protein levels in osteosarcoma cells after ELFN1-AS1 overexpression with or without LYPLA1 silencing. **(I, J)** Intracellular triglyceride and cholesterol levels in indicated osteosarcoma cells. **(K)** Oil Red O staining in indicated osteosarcoma cells. **(L)** CCK-8 assays showing cell proliferation under different treatments. **(M)** Transwell migration and invasion assays of U2OS cells under different treatments. Scale bar: 100 μm. **(N)** Cellular fractionation assay showing subcellular distribution of ELFN1-AS1 in U2OS cells. U6 and GAPDH are nuclear and cytoplasmic reference controls, respectively. **(O)** Actinomycin-D chase assay detecting accelerated LYPLA1 mRNA degradation upon ELFN1-AS1 knockdown in U2OS cells. **(P)** Proposed working model: ELFN1-AS1 stabilizes LYPLA1 mRNA and upregulates LYPLA1 to trigger lipid accumulation-associated phenotype, thereby promoting osteosarcoma proliferation and metastasis. All data are presented as mean ± SD. *P < 0.05, **P < 0.01, ***P < 0.001, ****P < 0.0001; ns, not significant.

Given the lipid metabolism-dependent features of the LMRL signature, we next investigated how ELFN1-AS1 modulates lipid phenotypes in osteosarcoma. Pearson correlation analysis revealed a positive association between ELFN1-AS1 and canonical lipogenic genes, including FASN, ACLY, ACC, SCD1, ACSL1 and CHKA (**Figure 10A**). Functionally, ELFN1-AS1 depletion reduced intracellular triglyceride, total cholesterol levels and lipid droplet abundance, alongside suppressed expression of core lipogenic genes in U2OS and 143B cells (**Figures 10B–D**; **Supplementary Figures 12A, B**), confirming ELFN1-AS1 promotes lipid accumulation-associated phenotype. To identify downstream effectors linking ELFN1-AS1 to lipid changes, we analyzed genes perturbed by in silico ELFN1-AS1 knockout. LYPLA1 exhibited the most prominent downregulation upon ELFN1-AS1 silencing (**Figure 10E**; **Supplementary Figure 12C**). Clinical data further demonstrated elevated LYPLA expression in osteosarcoma tissues, and high LYPLA1 levels correlated with inferior patient prognosis (**Figures 10F, G**).

Dual rescue assays were performed to validate the ELFN1-AS1/LYPLA1 regulatory axis. First, forward rescue experiments showed that LYPLA1 knockdown attenuated LYPLA1 upregulation triggered by ELFN1-AS1 overexpression (**Figure 10H**; **Supplementary Figure 12D**). ELFN1-AS1 overexpression robustly boosted lipogenic activity, whereas LYPLA1 silencing or treatment with orlistat (a selective FASN inhibitor) partially reversed this lipogenic phenotype (**Figures 10I–K**). To exclude non-specific cytotoxicity as a confounding factor, gradient CCK-8 proliferation assays were performed following 48 h orlistat treatment in 143B and U2OS cell lines (**Supplementary Figure 12E**). At the 10 μM working concentration with 48 h incubation used for all functional rescue assays, orlistat barely compromised the viability of OE-NC control cells yet exerted dose-dependent growth suppression in ELFN1-AS1-overexpressing cells, verifying its specific anti-lipogenic activity rather than broad toxic effects. Consistently, the enhanced proliferative, migratory and invasive capacities induced by ELFN1-AS1 overexpression were rescued by LYPLA1 knockdown or orlistat treatment (**Figures 10L, M**; **Supplementary Figures 12F–I**). Next, reciprocal rescue experiments were conducted to further establish the causal ELFN1-AS1/LYPLA1 relationship. We ectopically overexpressed LYPLA1 in ELFN1-AS1-knockdown osteosarcoma cells, and western blot results confirmed that LYPLA1 expression is restored in the background of ELFN1-AS1 knockdown (**Supplementary Figure 12J**). Functional assays revealed that exogenous LYPLA1 largely rescued the reduced lipid accumulation and impaired malignant phenotypes caused by ELFN1-AS1 silencing (**Supplementary Figures 12K-S**), firmly establishing LYPLA1 as an essential downstream mediator of ELFN1-AS1.

Having confirmed the causal ELFN1-AS1/LYPLA1 axis via dual rescue assays, we further dissected the underlying molecular mechanism. RT-qPCR and western blot data illustrated that LYPLA1 mRNA and protein levels declined upon ELFN1-AS1 knockdown, but increased significantly following ELFN1-AS1 overexpression (**Supplementary Figures 12T, J**; **Figure 10H**). Cellular fractionation assays demonstrated that ELFN1-AS1 predominantly localized to the cytoplasm (**Figure 10N**; **Supplementary Figure 12U**), indicating it may exert post-transcriptional regulatory functions. Actinomycin-D chase experiments further revealed faster LYPLA1 mRNA decay after ELFN1-AS1 silencing (**Figure 10O**; **Supplementary Figure 12V**), demonstrating that ELFN1-AS1 stabilizes LYPLA1 mRNA to elevate its expression. In summary, multi-dimensional correlation, biochemical and dual rescue experiments delineate the complete ELFN1-AS1/LYPLA1 regulatory cascade. Cytoplasmic ELFN1-AS1 elevates LYPLA1 abundance by stabilizing its mRNA, which drives lipid accumulation-associated phenotype and ultimately accelerates osteosarcoma proliferation, migration and invasion (**Figure 10P**).

## Discussion

Osteosarcoma remains a clinically challenging malignancy in which therapeutic progress has been constrained by profound biological heterogeneity, early metastatic dissemination, and limited improvement in outcomes for patients with recurrent, metastatic, or chemotherapy-refractory disease (2, 3, 24, 25). Lipid metabolic reprogramming has increasingly been recognized as a critical adaptive mechanism through which malignant cells fulfill bioenergetic, biosynthetic, and signaling requirements while withstanding hostile microenvironmental conditions (26, 27). Meanwhile, lncRNAs have emerged as versatile regulators of tumor biology, contributing to malignant processes such as proliferation, metastasis, immune remodeling, and therapeutic resistance (19, 28, 29). However, the prognostic and mechanistic relevance of LMRLs in osteosarcoma remains poorly defined. In this study, we integrated single-cell and bulk transcriptomic analyses to characterize lipid-metabolic heterogeneity and constructed a five-LMRL signature that stratified patients into prognostically and biologically distinct subgroups. Further validation identified ELFN1-AS1 as a clinically relevant lncRNA that promotes osteosarcoma progression through LYPLA1-dependent lipid accumulation-associated phenotype, highlighting a potential lncRNA-lipid metabolism axis for prognostic refinement and therapeutic exploration.

Altered lipid metabolism endows tumor cells with greater biological flexibility, allowing them to adjust growth-related signaling, maintain invasive and metastatic traits, and withstand therapeutic or microenvironmental stress (9, 27, 30). Key regulators of lipid metabolism, particularly those involved in fatty acid, cholesterol, and sphingolipid pathways, are closely associated with tumor initiation and progression, making them attractive candidates for metabolic intervention in cancer therapy (31–33). Recent studies across diverse solid tumors have demonstrated that lipid metabolic remodeling carries both biological and translational significance. In male breast cancer, enhanced fatty acid pathway activity and FASN upregulation point to a tumor-cell metabolic dependency (34), whereas in early-stage lung cancer, glycerophospholipid dysregulation has been translated into a circulating nine-lipid signature for noninvasive diagnosis (35). In osteosarcoma, emerging studies have demonstrated that targeting lipid metabolic regulators, including FASN and FABP4, can suppress tumor progression by disrupting fatty acid metabolism and downstream PI3K/AKT or MAPK signaling, highlighting lipid metabolic reprogramming as a potential therapeutic vulnerability (36, 37). Extending these observations, our single-cell analysis revealed that lipid metabolic activity was unevenly distributed across malignant osteosarcoma subpopulations rather than uniformly increased throughout the tumor compartment. Moreover, projection of lipid metabolism scores along pseudotime suggested that lipid remodeling accompanied specific malignant cell-state transitions, indicating that metabolic plasticity may be coordinated with transcriptional evolution. Notably, high-lipid-metabolism OS cells showed more pronounced communication with MSCs, fibroblasts, endothelial cells, and pericytes, involving collagen-integrin, VEGF, MIF, FN1, SPP1, and chemokine-related signaling programs. These findings suggest that dysregulated lipid metabolism may participate not only in intrinsic malignant cell adaptation but also in tumor-microenvironment crosstalk. Functionally, our rescue experiments using orlistat, a selective FASN inhibitor that specifically blocks *de novo* lipogenesis, verified that targeted suppression of lipogenic activity effectively attenuates the oncogenic potency of the ELFN1-AS1/LYPLA1 axis driven lipid accumulation-associated phenotype. Collectively, the LMRL-based prognostic signature established in this study enables precise patient risk stratification, and targeted intervention against lipid metabolic dysfunction represents a promising combinatorial therapeutic strategy for high-risk osteosarcoma patients.

LncRNAs are increasingly being incorporated into the regulatory map of lipid metabolism, where they may influence lipid accumulation, fatty acid utilization, lipid peroxidation, and metabolism-dependent therapeutic responses (38, 39). In breast cancer, lncROPM stabilizes PLA2G16 mRNA to enhance phospholipid metabolism and arachidonic acid production, thereby activating PI3K/AKT, Wnt/β-catenin, and Hippo/YAP signaling to sustain cancer stemness and chemoresistance (40). In lung cancer, extracellular vesicle-transferred lncRNA ROLLCSC enhances the plasticity and metastatic potential of non-stem cancer cells by functioning as a ceRNA for miR-5623-3p and miR-217-5p, thereby activating lipid metabolism-related programs that promote metastatic colonization (41). In osteosarcoma, although lipid-focused lncRNA mechanisms remain less extensively characterized, several studies have already suggested that lncRNAs can reshape metabolism-dependent malignant behavior. Previous studies have identified the LMRLs SNHG17 and LINC00837 as aberrantly upregulated in osteosarcoma tissues and cells, where they function as independent prognostic indicators (42). In our experimental analyses, ELFN1-AS1 emerged as a LMRL that was highly expressed in osteosarcoma tissues and cells and associated with more advanced disease and unfavorable prognosis. Functionally, ELFN1-AS1 depletion impaired lipid metabolic activity and suppressed osteosarcoma proliferation and progression *in vitro* and *in vivo*. Mechanistically, our preliminary data suggest that ELFN1-AS1 promotes malignant phenotypes through LYPLA1-dependent lipid accumulation-associated phenotype. This finding is consistent with a recent study showing that RPARP-AS1 enhances lipid metabolism through FABP4, MAGL, SCD1, and Akt/mTOR signaling, thereby promoting tumor cell proliferation and survival (43). Together, these observations suggest that lncRNA-governed lipid metabolic dysfunction constitutes a critical regulatory mechanism that facilitates malignant progression of osteosarcoma.

The tumor immune microenvironment is a decisive modifier of tumor progression and therapeutic response (44–46), while recent studies have suggested that lncRNA-based signatures can capture immune infiltration patterns and immunotherapy-related features across cancers, indicating that lncRNAs may reflect not only tumor-intrinsic transcriptional states but also broader immune ecological variation (47–50). In parallel, accumulating evidence suggests that immune-contexture differences may partially underlie the prognostic heterogeneity observed among patients with osteosarcoma (51–53). Our purely computational analyses identified correlations between the LMRL risk signature and distinct immune, genomic and predicted therapeutic traits, yet these associations lack validation via clinical tissue staining or immunocompetent animal models. Low-risk patients displayed elevated ImmuneScore and ESTIMATE Score, alongside higher enrichment of natural killer T cells, activated CD8^+^T cells, macrophages and neutrophils, which hints at a potentially immune-reactive microenvironment with abundant immune cell infiltration. Concordantly, multiple immune functional signatures (APC co-stimulation, cytolytic activity, checkpoint activity, T-cell co-inhibition) were also enriched in low-risk tumors, tentatively pointing to more robust intratumoral immune activation in this subgroup. In contrast, high-risk tumors possessed elevated tumor purity and relatively sparse immune cell infiltration, accompanied by higher TMB and upregulated expression of immune inhibitory molecules including CD276, HAVCR2, LAG3, LGALS9 and TNFRSF14. This computational pattern implies the high-risk subgroup may represent an aggressive tumor phenotype featuring limited immune cell recruitment but prominent immune exhaustion and immunosuppressive signaling. In silico drug sensitivity prediction further revealed subgroup-specific disparities in estimated IC_50_ values: high-risk patients were predicted to respond better to vorinostat, axitinib, sorafenib, linsitinib, MK-2206 and LY2109761, while low-risk patients exhibited lower predicted IC_50_ for 5-fluorouracil, trametinib and ruxolitinib. All immune and therapeutic differences above stem only from bioinformatic inference and cannot be treated as definitive functional evidence. Collectively, these exploratory computational correlations suggest the LMRL signature reflects multidimensional osteosarcoma heterogeneity via computationally inferred immune features, genomic alterations and predicted therapeutic vulnerabilities.

However, several limitations should be acknowledged in this study. First, training and internal validation cohorts were both sourced from the TARGET-OS public dataset; only internal model evaluation was performed without independent external cohort verification. Large prospective multicenter cohorts with standardized RNA-seq protocols are required to validate the signature’s discrimination, calibration, generalizability and clinical value. Second, although our functional assays verified that ELFN1-AS1 promotes lipid accumulation-associated phenotypes and osteosarcoma progression via upregulating LYPLA1, the detailed regulatory machinery remains incompletely defined. Specifically, our current results demonstrated that cytoplasmic ELFN1-AS1 stabilizes LYPLA1 mRNA and suppresses its degradation, thereby elevating LYPLA1 expression. However, the intermediate molecules that bridge ELFN1-AS1 and LYPLA1 mRNA, including potential RNA-binding proteins or competitive endogenous RNAs, remain uncharacterized. Further investigations incorporating RIP-seq, RNA pull-down, nascent RNA detection, and promoter activity validation are therefore warranted to fully dissect the post-transcriptional regulatory network of ELFN1-AS1. These mechanistic explorations were not included in the current revision and will be systematically addressed in our future follow-up studies. Additionally, systematic lipid profiling such as lipidomics and isotope tracing was absent in our work, restricting full interpretation of lipid metabolic shifts. Third, although the subcutaneous xenograft model allows reproducible tumor growth and exploratory assessment of pulmonary dissemination, it does not recapitulate the native bone microenvironment or the complete metastatic cascade of osteosarcoma. Therefore, the reduced pulmonary metastatic foci observed after ELFN1-AS1 depletion should be interpreted within this model context and require further validation in orthotopic osteosarcoma models. Moreover, *in vivo* assays used BALB/c nude mice with defective adaptive immunity, failing to reconstruct intact anti-tumor immune microenvironment. All immune-related results are merely computational predictions; clinical IHC or multiplex-IF validation could not be finished in this revision due to insufficient annotated patient samples and will be pursued in follow-up work. Furthermore, while the ELFN1-AS1/LYPLA1 axis holds therapeutic potential, targeted lncRNA delivery to osteosarcoma remains challenging. Future work will test extracellular vesicles and lipid nanocarriers, and evaluate combinatorial effects with chemo-, targeted, or immunotherapy.

## Conclusion

In conclusion, this study integrated single-cell transcriptomics, bulk RNA-seq, prognostic modeling, virtual knockout screening and experimental validation to characterize LMRLs in osteosarcoma. We revealed heterogeneous lipid metabolic activity across malignant cell states and established a five-LMRL signature that stratified patients by prognosis, immune landscape, mutational features, and predicted therapeutic response. Notably, ELFN1-AS1 was identified as a key oncogenic LMRL driving osteosarcoma progression via triggering LYPLA1-dependent lipid accumulation-associated phenotype. These findings expand the current understanding of lncRNA-mediated metabolic regulation and highlight the ELFN1-AS1/LYPLA1 regulatory cascade as a potential biomarker and therapeutic target. Targeted inhibition of *de novo* lipogenesis using selective FASN inhibitors such as orlistat provides a feasible metabolic intervention strategy for precision treatment of high-risk osteosarcoma.

## Data Availability

The datasets presented in this study can be found in online repositories. The names of the repository/repositories and accession number(s) can be found in the article/**Supplementary Material**.
