# Supplementary material for: Integrative single-cell and bulk transcriptomics uncovers a lipid metabolism-related lncRNA signature alongside the ELFN1-AS1/LYPLA1 axis driving osteosarcoma progression
Source: Front Immunol. 2026 Sep 11;17:1945005. doi: 10.3389/fimmu.2026.1945005 (PMC13612571; doi:10.3389/fimmu.2026.1945005)
Supplement: Supplementary file 1 [file DataSheet1.pdf]

Original WB data

After membrane transfer, the region corresponding to 25-35 kDa was excised and incubated with a LYPLA1 antibody. Protein bands were visualized using enhanced chemiluminescence.

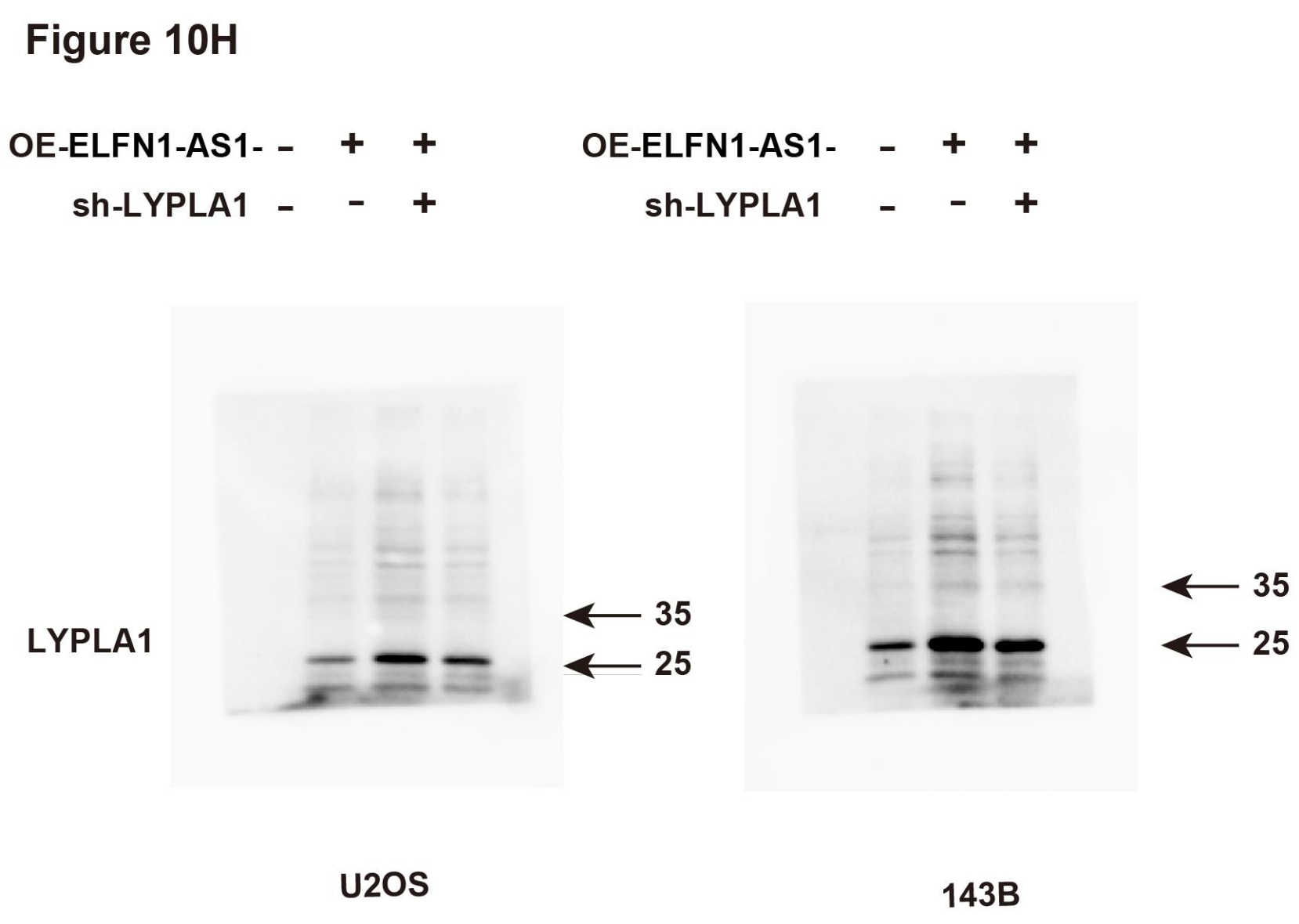

After membrane transfer, the region corresponding to 35-40 kDa was excised and incubated with a GAPDH antibody. Protein bands were visualized using enhanced chemiluminescence.

Figure 10H

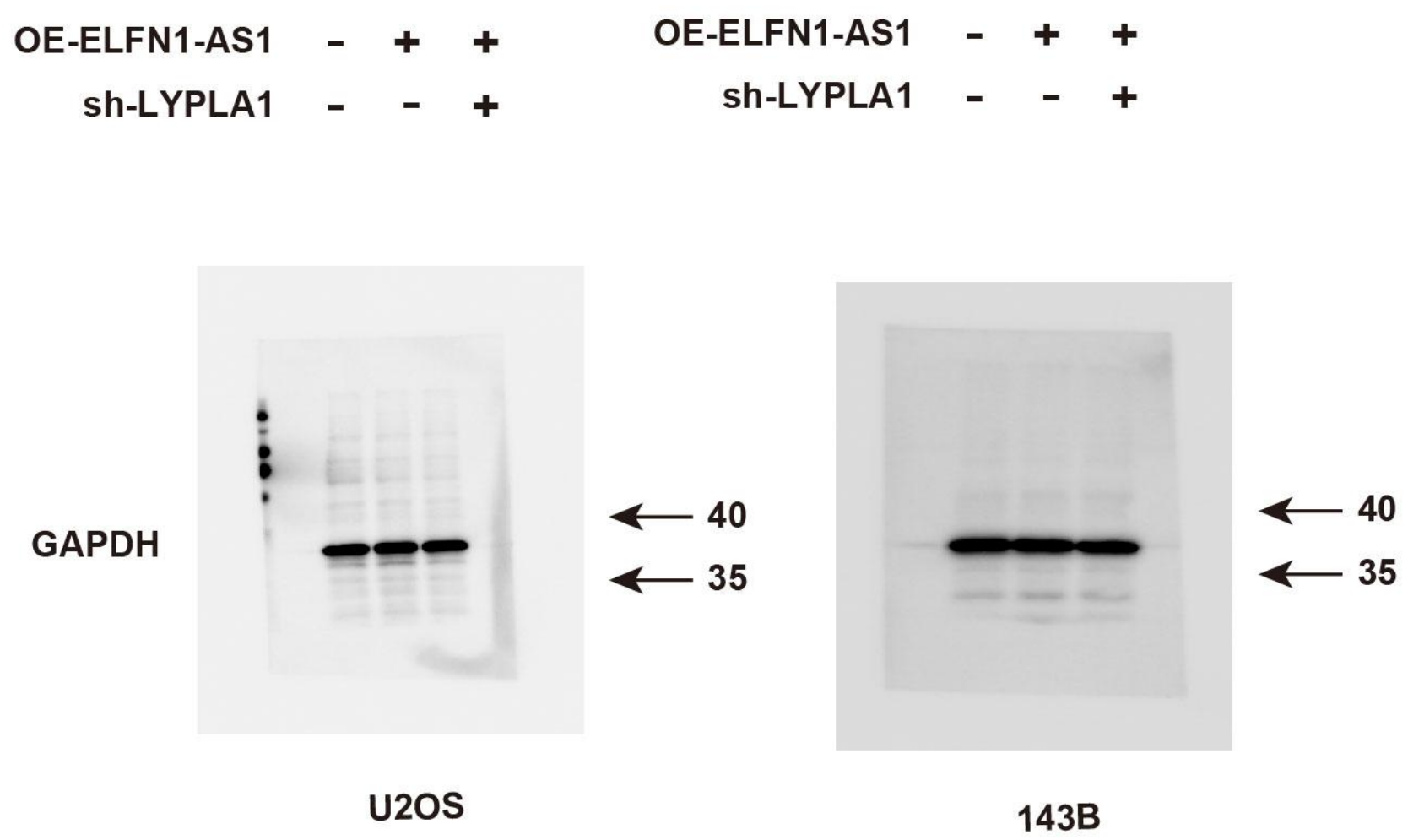

.

After membrane transfer, the region corresponding to 25-35 kDa was excised and incubated with a LYPLA1 antibody. Protein bands were visualized using enhanced chemiluminescence.

Figure S12J

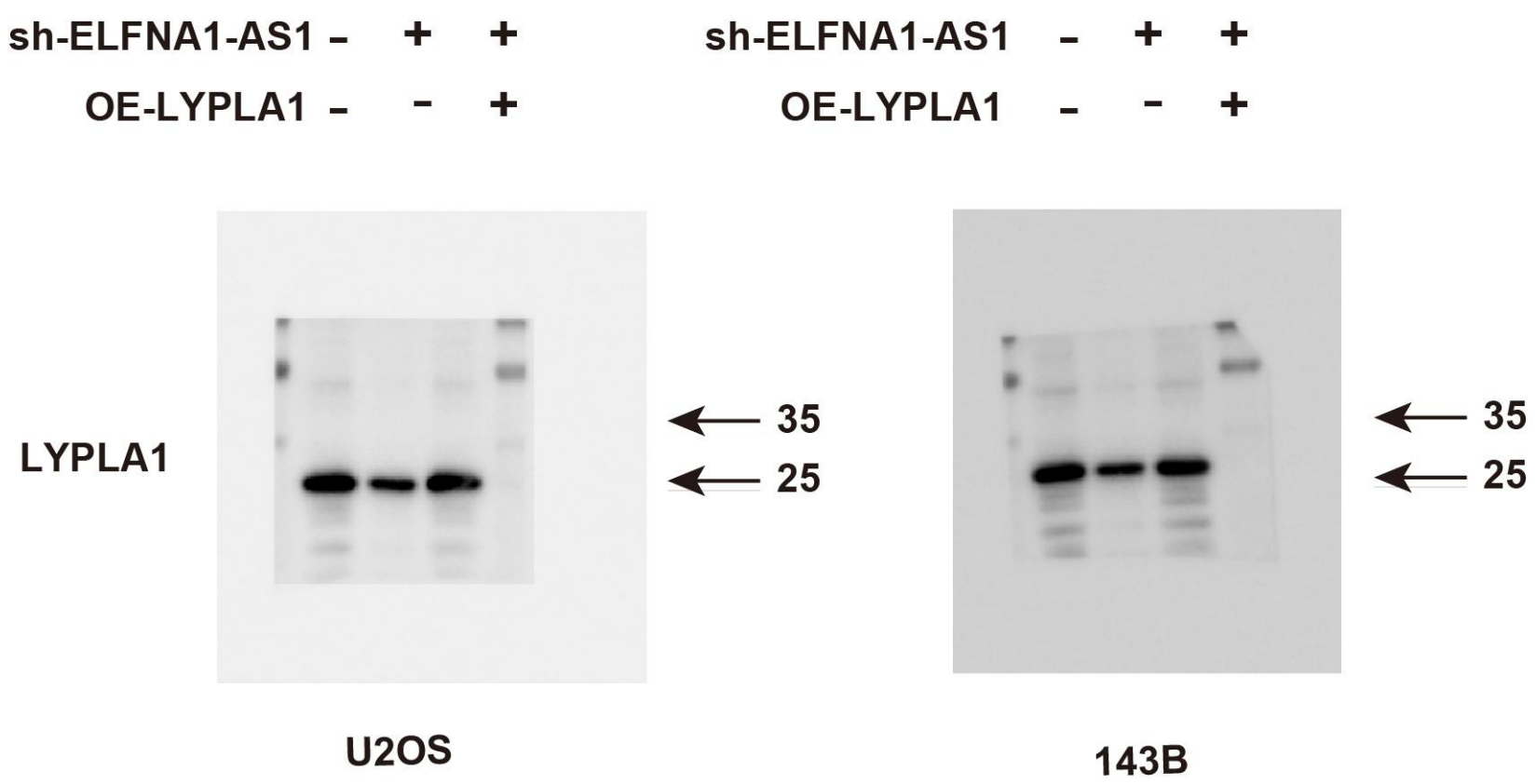

After membrane transfer, the region corresponding to 35-40 kDa was excised and incubated with a GAPDH antibody. Protein bands were visualized using enhanced chemiluminescence.

Figure S12J

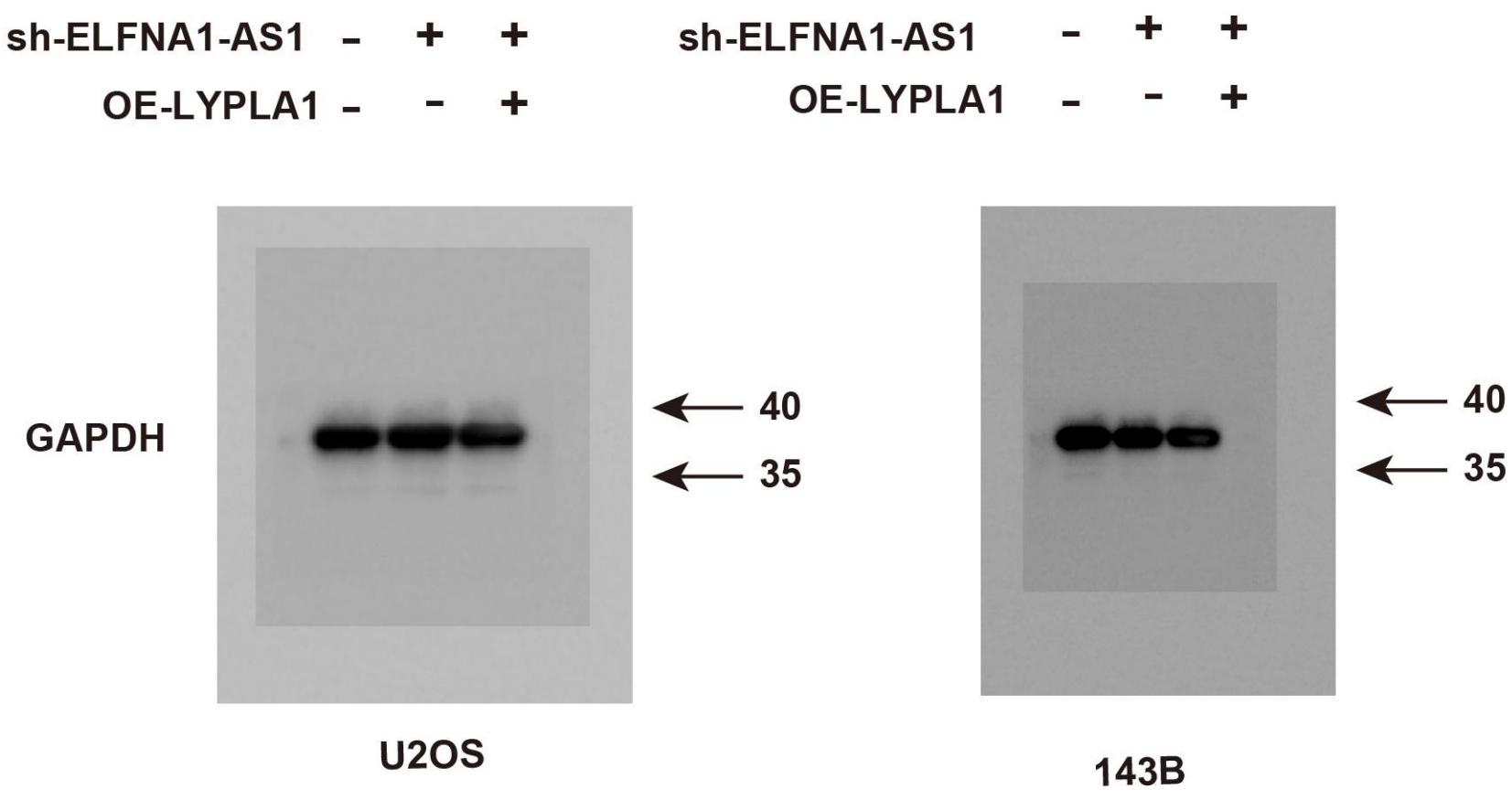
sh-ELFNA1-AS1 - + +
OE-LYPLA1 - - +
sh-ELFNA1-AS1 - + +
OE-LYPLA1 - - +
GAPDH
40
35
40
35
U2OS
143B
