# Supplementary material for: Integrative single-cell and bulk transcriptomics uncovers a lipid metabolism-related lncRNA signature alongside the ELFN1-AS1/LYPLA1 axis driving osteosarcoma progression
Source: Front Immunol. 2026 Sep 11;17:1945005. doi: 10.3389/fimmu.2026.1945005 (PMC13612571; doi:10.3389/fimmu.2026.1945005)
Supplement: Supplementary file 2 [file DataSheet2.docx]

**Supplementary figures**

**Figure S1.** Single-cell characterization of cellular composition, intercellular communication, and lipid metabolic heterogeneity in osteosarcoma. **(A)** Dot plot of marker gene expression across 25 cell clusters. **(B)** Relative cell-type composition across individual osteosarcoma samples. **(C-D)** OS-centered communication networks showing outgoing signaling from OS cells (C) and incoming signaling toward OS cells (D). **(E)** UMAP projection of lipid metabolism AUCell scores across 11 annotated cell types. **(F)** UMAP visualization of all cells stratified into high- and low-lipid-metabolism groups based on AUCell scores.

**Figure S2.** Single-cell delineation of osteosarcoma subclusters and lipid metabolism-associated transcriptional heterogeneity. **(A)** CNV heatmap showing greater chromosome-scale alterations in the three OS cell populations than in reference immune cells. **(B)** All three OS populations exhibited significantly higher CNV scores than the reference cells. **(C)** UMAP visualization of seven clusters identified after reclustering the extracted OS cells. **(D)** Dot plot of marker gene expression across seven OS cell clusters. **(E)** Relative composition of OS cell subclusters across individual osteosarcoma samples. **(F)** Distribution of lipid metabolism AUC scores among seven malignant OS cell subclusters. **(G)** Heatmap of DEGs between high- and low-lipid-metabolism OS cells. **(H)** GO enrichment analysis of DEGs between high- and low-lipid-metabolism OS cells. *****P* < 0.0001.

**Figure S3.** Pseudotemporal dynamics of lineage-associated gene expression and lipid metabolic activity in osteosarcoma cells. **(A-B)** Expression trajectories of ACAN, COL1A1, COL2A1, COL3A1, RUNX2, and SOX9, colored by OS subcluster **(A)** and pseudotime progression **(B)**. **(C)** Subcluster-specific changes in lipid metabolism AUC scores along pseudotime.

**Figure S4.** Intercellular communication differences in malignant OS cells with high and low lipid metabolism scores. **(A-D)** OS-centered communication networks showing incoming and outgoing signaling in the high- **(A-B)** and low-lipid-metabolism **(C-D)** groups. **(E-F)** Bubble plots illustrating ligand-receptor interactions from surrounding cell populations to OS cells in the high- **(E)** and low-lipid-metabolism **(F)** groups

**Figure S5.** Construction of a LMRL signature for osteosarcoma prognosis in the training cohort. **(A-C)** Risk-score distribution, survival status, and five-lncRNA expression profiles in the training cohort.

**Figure S6.** Validation of the LMRL risk model. **(A-B)** Survival status and five-lncRNA expression profiles in the total sample group. **(C)** Kaplan-Meier analysis showing significantly poorer overall survival in the high-risk group within the total sample group (*P*=0.0003). **(D)** Time-dependent ROC curves of the prognostic signature for predicting 1-, 3-, and 5-year overall survival in the total sample group (AUCs = 0.833, 0.804, and 0.809, respectively). **(E)** ROC curves comparing the prognostic performance of the risk score and clinical variables in the total sample group (AUCs: risk score, 0.809; age, 0.455; gender, 0.424; metastatic status, 0.704). **(F-G)** Univariate **(F)** and multivariable **(G)** Cox regression analyses of the risk score and clinical variables in the total sample group.

**Figure S7.** Differential transcriptional profiles and functional enrichment of osteosarcoma risk groups. **(A)** Volcano plot of DEGs between the low- and high-risk osteosarcoma groups. **(B)** Heatmap of DEGs between the low- and high-risk osteosarcoma groups, with red indicating higher expression and blue indicating lower expression. **(C)** GO enrichment analysis of DEGs between the low- and high-risk osteosarcoma groups. **(D)** KEGG pathway enrichment of DEGs between the low- and high-risk osteosarcoma groups.

**Figure S8.** Functional, immune, genomic, and therapeutic heterogeneity between LMRL risk subgroups. **(A)** Comparison of tumorpurity between the low- and high-risk groups. **(B)** Boxplot showing significantly higher TMB in the high-risk group than in the low-risk group. **(C)** Correlation between risk score and TMB. **(D)** Comparison of predicted sensitivity to each compound between the low- and high-risk groups.

**Figure S9.** Prognostic performance of individual LMR-lncRNAs in osteosarcoma. **(A-E)** Overall survival according to high versus low expression of AL133410.1 **(A)**, AL596247.1 **(B)**, IL10RB-DT **(C)**, NECTIN3-AS1 **(D)**, and ELFN1-AS1 **(E)**. **(F-J)** Time-dependent ROC analyses of AL133410.1 **(F)**, AL596247.1 **(G)**, IL10RB-DT **(H)**, NECTIN3-AS1 **(I)**, and ELFN1-AS1 **(J)** for 1-, 3-, and 5-year survival prediction.

**Figure S10.** Single-cell distribution of ELFN1-AS1 in osteosarcoma. **(A)** UMAP feature plot showing ELFN1-AS1 expression across all annotated cells. **(B)** UMAP feature plot showing ELFN1-AS1 expression across OS cells.

**Figure S11.** Functional validation of ELFN1-AS1 as an oncogenic driver of osteosarcoma progression *in vitro* and *in vivo*. **(A)** CCK-8 assays showing reduced cell viability in ELFN1-AS1-silenced 143B cells over three days. **(B)** EdU assays demonstrating fewer proliferative cells in sh-ELFN1-AS1#1 and sh-ELFN1-AS1#2 groups compared with sh-NC in 143B cells. Scale bar: 100 μm. **(C)** Transwell migration and invasion assays showing impaired migratory and invasive abilities of 143B cells after ELFN1-AS1 knockdown. Scale bar: 100 μm. All data are presented as mean ± SD. ****P* < 0.001, *****P* < 0.0001.

**Figure S12.** ELFN1-AS1 drives osteosarcoma malignant progression via activating LYPLA1-mediated lipid accumulation-associated phenotype. **(A‑B)** RT‑qPCR analysis of key canonical lipid metabolism genes expression in U2OS and 143B cells with ELFN1-AS1 knockdown. **(C)** RT‑qPCR analysis of candidate target genes expression in 143B cells with ELFN1-AS1 knockdown. **(D)** RT‑qPCR confirmation of ELFN1-AS1 overexpression efficiency in U2OS and 143B cells. **(E)** Cell viability of U2OS and 143B cells treated with gradient concentrations of orlistat. **(F)** CCK‑8 assays showing cell proliferation in 143B cells under different treatments. **(G)** Quantification of migration and invasion capacity under LYPLA1 knockdown or orlistat treatment upon ELFN1-AS1 overexpression in U2OS and cells. **(H‑I)** Transwell migration and invasion assays of 143B cells under different treatments. **(J)** Western blot analysis of LYPLA1 protein levels in osteosarcoma cells after ELFN1-AS1 knockdown with or without LYPLA1 overexpression. **(K-L)** Intracellular triglyceride and cholesterol levels in indicated osteosarcoma cells. **(M)** Oil Red O staining in indicated osteosarcoma cells. **(N-O)** CCK‑8 proliferation assays demonstrating that ectopic LYPLA1 overexpression reverses the growth suppression caused by ELFN1‑AS1 knockdown in U2OS and 143B cells. **(P-S)** Transwell migration and invasion assays of U2OS and 143B cells after LYPLA1 restoration under ELFN1‑AS1 knockdown background. **(T)** RT‑qPCR measurement of LYPLA1 mRNA levels in ELFN1‑AS1 overexpression and knockdown osteosarcoma cells. **(U)** Cellular fractionation assay showing subcellular distribution of ELFN1‑AS1 in 143B cells. U6 and GAPDH are nuclear and cytoplasmic reference controls, respectively. **(V)** Actinomycin‑D chase assay assessing LYPLA1 mRNA decay rate in sh‑NC and sh‑ELFN1‑AS1 143B cells. Scale bar: 100 μm. All data are presented as mean ± SD. **P* < 0.05, ***P* < 0.01, ****P* < 0.001, *****P* < 0.0001; ns, not significant.
