# Supplementary material for: Integrative single-cell and bulk transcriptomics uncovers a lipid metabolism-related lncRNA signature alongside the ELFN1-AS1/LYPLA1 axis driving osteosarcoma progression
Source: Front Immunol. 2026 Sep 11;17:1945005. doi: 10.3389/fimmu.2026.1945005 (PMC13612571; doi:10.3389/fimmu.2026.1945005)
Supplement: Supplementary file 3 [file SupplementaryFile1.docx]

**Supplementary materials and methods**

**Copy number variation** **(CNV) analysis**

To further support the malignant identity of the annotated OS populations, large-scale CNV patterns were inferred from single-cell transcriptomic data using the inferCNV R package, with TILs and myeloid cells serving as non-malignant reference populations. Genes were ordered according to their chromosomal positions in the hg38 genome, and CNV signals were inferred relative to the reference-cell expression baseline. The analysis was performed with cutoff = 0.1, cluster_by_groups = TRUE, and denoise = TRUE, using the sample-level analysis mode without HMM-based discrete CNV calling.

**Cell-cell communication analysis**

Potential intercellular signaling among annotated osteosarcoma cell populations was investigated using the CellChat R package according to its standard workflow. For this analysis, only malignant OS cells were stratified into lipid-metabolism-high and -low states, whereas non-malignant populations retained their original cell-type identities; all populations were subsequently incorporated into the same CellChat communication framework. Normalized gene-expression data were integrated with CellChatDB.human to infer communication events from the coordinated expression of ligands, receptors, and associated cofactors. Overexpressed signaling genes and ligand–receptor pairs were identified and projected onto the human protein–protein interaction network, after which communication probabilities were calculated. Interactions involving cell populations containing fewer than 10 cells were excluded. The inferred signals were subsequently summarized at the pathway level, and network centrality analysis was used to characterize the outgoing and incoming signaling roles of individual cell populations.

**Differential expression analysis**

Differentially expressed genes (DEGs) between OS cells with high and low lipid metabolism activity were identified using the limma R package, which performs gene-wise linear modeling within an empirical Bayes framework. Genes satisfying both∣log2FC∣>1 and *P*<0.05 were considered statistically significant DEGs.

**Functional enrichment analysis**

Functional enrichment analysis of the differentially expressed genes was performed using the clusterProfiler R package. Gene Ontology (GO) analysis covered the biological process, cellular component, and molecular function categories, while Kyoto Encyclopedia of Genes and Genomes (KEGG) analysis was conducted to identify significantly overrepresented pathways. Enrichment terms with *P* < 0.05 were considered statistically significant.

**Pseudotime trajectory analysis**

Pseudotemporal trajectories of OS cells were reconstructed using the Monocle 2 R package, which applies reversed graph embedding to resolve complex cellular state transitions. Raw UMI count matrices derived from the Seurat object were used as input, and genes detected in at least 10 cells were retained. A CellDataSet object was generated using the negative binomial expression model, with the lower detection limit set to 0.5, followed by estimation of size factors and gene dispersions. Ordering genes were selected according to a mean expression of at least 0.1 and an empirical dispersion no lower than the fitted dispersion. Dimensionality reduction was conducted using DDRTree with two components, while sample-specific variation was adjusted using the sample identity as a residual covariate. Cells were subsequently arranged along the inferred trajectory using orderCells() and visualized according to pseudotime, cellular state, and OS subpopulation. The dynamic expression patterns of representative osteoblastic and chondroblastic markers, together with lipid metabolism AUC scores, were examined across pseudotime. Pseudotime-associated genes were identified using a spline-based differential expression model, and branch-dependent transcriptional changes were further evaluated by branch expression analysis modeling at the first branch point.

**Evaluation of the prognostic signature**

The prognostic performance of the lncRNA signature was assessed in the training, test, and overall cohorts. Risk-score distributions, survival-status plots, and model-lncRNA expression heatmaps were generated to illustrate outcome patterns across the risk strata. Differences in overall survival between the high- and low-risk groups were evaluated using Kaplan–Meier analysis with the log-rank test through the survival and survminer R packages. Time-dependent receiver operating characteristic curves and the corresponding areas under the curve were calculated using the timeROC package to assess predictive accuracy. Furthermore, univariable and multivariable Cox regression analyses incorporating the risk score and available clinical characteristics were performed to determine whether the signature served as an independent prognostic indicator for OS.

**Nomogram construction and validation**

A prognostic nomogram integrating the LMRL risk score with age, gender, and metastatic status was developed using the rms R package to estimate individualized 1-, 3-, and 5-year overall survival probabilities. Model discrimination was evaluated using time-dependent receiver operating characteristic curves and the corresponding area under the curve values, whereas calibration curves were generated to assess agreement between the predicted and observed survival outcomes.

**Immune landscape analysis**

Single-sample gene set enrichment analysis (ssGSEA), implemented using the GSVA and GSEABase R packages, was performed to quantify sample-specific enrichment of immune cell populations and immune-related functional signatures. The resulting scores were compared between the high- and low-risk groups using the limma package. In parallel, the ESTIMATE algorithm was applied to calculate StromalScore, ImmuneScore, and ESTIMATEScore and to infer tumor purity. Differences in immune checkpoint gene expression between the two risk groups were further assessed using a two-sided Wilcoxon rank-sum test.

**Drug sensitivity analysis**

Model-predicted drug responses were estimated for the 84 TARGET-OS samples using the “calcPhenotype” function in the oncoPredict R package (version 1.2) under R version 4.3.3. The TARGET-OS expression matrix was normalized as log2(TPM + 1), and the preprocessed expression and pharmacological-response data from the Genomics of Drug Sensitivity in Cancer 2 (GDSC2) cell-line panel were used as the training datasets. For each compound, oncoPredict constructed a ridge-regression model based on GDSC2 cell-line expression and drug-response data and applied the fitted model to the TARGET-OS samples. Empirical-Bayes batch correction was used (batchCorrect = “eb”), drug-response phenotypes were power-transformed, the lowest-varying 20% of genes were removed, and at least 10 cell lines with available response data were required for each compound-specific model. Predicted response values were compared between the predefined high- and low-risk groups using a two-sided Wilcoxon rank-sum test. *P* values across all evaluated compounds were adjusted using the Benjamini-Hochberg false-discovery-rate procedure, with an adjusted *P* value below 0.05 considered statistically significant.

**Somatic mutation analysis**

Somatic mutation profiles were analyzed separately in the high- and low-risk groups using the maftools R package. Waterfall plots were generated to summarize mutation types, frequencies, and recurrently altered genes, enabling comparison of the mutational landscapes between the two risk subgroups.

**Cell lines and drugs**

The human osteosarcoma cell lines 143B, U2OS, MG63, SaOS-2, and MNNG/HOS were used alongside the nonmalignant osteoblastic cell line hFOB1.19 and the human embryonic kidney cell line 293T, all of which were obtained from the American Type Culture Collection (ATCC, Manassas, VA, USA). Cells were routinely propagated in Dulbecco’s modified Eagle medium (DMEM; Cellmax, Beijing, China) containing 10% fetal bovine serum (FBS; Cellmax, Beijing, China) and 1% penicillin-streptomycin (Thermofisher, USA) in a humidified atmosphere with 5% CO₂. Osteosarcoma cells and 293T cells were maintained at 37°C, whereas hFOB1.19 cells were cultured at 34°C. Orlistat (MedChemExpress, USA), a selective FASN inhibitor that specifically blocks de novo lipogenesis, was dissolved in DMSO to prepare a 10 mM stock solution. For functional rescue experiments, osteosarcoma cells were treated with 10 μM orlistat for 48 h. Gradient concentration screening combined with 48 h CCK-8 assays confirmed that this working dose exerted specific anti-lipogenic effects with negligible non-specific cytotoxicity, and equal DMSO was used as the vehicle control.

**Plasmids reagents and cell transfection**

Tsingke Biotechnology (Beijing, China) generated all lentiviral constructs used in this study. ELFN1-AS1 overexpression and control plasmids were prepared in the pCDH backbone, whereas sh-NC, sh-ELFN1-AS1#1, sh-ELFN1-AS1#2, and sh-LYPLA1 were carried by the pLKO.1 vector. Recombinant lentiviruses were produced in 293T packaging cells and introduced into OS cells. Transduced cells were maintained under puromycin selection at 2 μg/mL for 5–7 days, after which the surviving populations were expanded for subsequent experiments. The efficiency of gene overexpression or knockdown was experimentally confirmed. The shRNA sequences were as follows: sh-ELFN1-AS1#1, GCATCTAATCCACCTGCAGAA; sh-ELFN1-AS1#2, CATTCTGGACTCCTCTCTTTC; sh-LYPLA1, CGGTGGTGCTAATAGAGATAT.

**Western blot assay**

Protein expression was examined by Western blotting, for which osteosarcoma cells and tissue specimens were lysed in RIPA buffer supplemented with phenylmethylsulfonyl fluoride and phosphatase inhibitors. After equal amounts of protein had been denatured in 5× SDS loading buffer at 100°C for 10 min, the samples were resolved by SDS-PAGE and electrotransferred onto 0.45-μm PVDF membranes. The membranes were blocked with 5% non-fat milk and then incubated with the indicated primary antibodies targeting LYPLA1 (Proteintech, Wuhan, China) and GAPDH (Proteintech, Wuhan, China) at 4°C overnight, followed by horseradish peroxidase-conjugated secondary antibodies for 1 h at room temperature. Immunoreactive bands were developed with enhanced chemiluminescence reagents and recorded using a Bio-Rad imaging system.

**Cell viability assay**

Osteosarcoma cells from the indicated experimental groups were plated in 96-well plates at 2×10^3 cells per well. At the designated time points, 10 μL of Cell Counting Kit-8 reagent (Yeasen, Shanghai, China) was added to each well, followed by incubation at 37°C for 2 h. The resulting colorimetric signal was measured at 450 nm using a microplate reader, and the absorbance values were used to evaluate changes in cell viability and generate growth curves.

**Colony formation assays**

Long-term proliferative capacity was assessed by culturing 1,000 osteosarcoma cells from each experimental group into 6-well plates. After 14 days of culture, the resulting colonies were gently washed with PBS, preserved in 4% paraformaldehyde for 20 min, and stained with 0.1% crystal violet for an additional 20 min. Excess stain was removed before the plates were air-dried and photographed, after which colony numbers were quantified using ImageJ software.

**Transwell assay**

Uncoated and Matrigel-coated Transwell inserts with 8.0-μm pores (Corning, NY, USA) were used to distinguish the migratory and invasive capacities of osteosarcoma cells. Equal numbers of osteosarcoma cells (4 × 10^3 cells) were placed in the upper compartment in serum-free DMEM, while medium containing 20% fetal bovine serum was added to the lower well to establish a chemotactic gradient. After incubation for 24 h at 37°C, cells remaining on the upper side of the membrane were removed, whereas those that had traversed the membrane were fixed with 4% paraformaldehyde and stained with crystal violet. The stained cells were photographed under a light microscope and quantified in randomly selected fields using ImageJ software.

5-**Ethynyl-20-deoxyuridine (EdU) assay**

Cell proliferation was assessed using an EdU imaging kit (Beyotime, Shanghai, China) according to the manufacturer’s instructions. Osteosarcoma cells were incubated with 50 μM EdU for 2 h at 37°C, fixed with 4% paraformaldehyde, permeabilized with Triton X-100, and subjected to the click-labeling reaction. Nuclei were counterstained with Hoechst 33342. Images were acquired using a fluorescence microscope, and the proliferation rate was calculated as the percentage of EdU-positive cells among total cells using ImageJ software.
