# Supplementary material for: Integrative single-cell and bulk transcriptomics uncovers a lipid metabolism-related lncRNA signature alongside the ELFN1-AS1/LYPLA1 axis driving osteosarcoma progression
Source: Front Immunol. 2026 Sep 11;17:1945005. doi: 10.3389/fimmu.2026.1945005 (PMC13612571; doi:10.3389/fimmu.2026.1945005)
Supplement: Supplementary file 4 [file Table2.docx]

**Supplementary Table 2.** Association of ELFN1-AS1expression with clinicopathological characteristics in patients with osteosarcoma

|  |  | ELFN1-AS1 expression | | | |  |
| --- | --- | --- | --- | --- | --- | --- |
| Characteristics | N | Low | | High | | *P* value |
|  | 32 | N=16（50.0%） | | N=16（50.0%） | |  |
| Age(years) |  |  |  |  |  | 0.1489 |
| < 18 | 19 | 7 | | 12 | |  |
| ≥ 18 | 13 | 9 | | 4 | |  |
| Gender |  |  |  |  |  | 0.7224 |
| Male | 18 | 8 | | 10 | |  |
| Female | 14 | 8 | | 6 | |  |
| site |  |  |  |  |  | 0.4083 |
| Tibia | 10 | 4 | | 6 | |  |
| Femur | 16 | 9 | | 7 | |  |
| Humerus | 3 | 2 | | 1 | |  |
| Fibula | 2 | 0 | | 2 | |  |
| Pelvis | 1 | 1 | | 0 | |  |
| Tumor size(cm) |  |  | |  | | 0.0732 |
| ≤ 8 | 14 | 10 | | 4 | |  |
| > 8 | 18 | 6 | | 12 | |  |
| Histological subtype |  |  |  |  |  | 0.9006 |
| Osteoblastic | 18 | 8 | | 10 | |  |
| Chondroblastic | 5 | 3 | | 2 | |  |
| Fibroblastic | 4 | 2 | | 2 | |  |
| parosteal | 2 | 2 | | 1 | |  |
| Others | 3 | 1 | | 2 | |  |
| Metastasis |  |  |  |  |  | **0.0285** |
| Yes | 12 | 3 | | 9 | |  |
| No | 20 | 13 | | 7 | |  |
| Response to chemotherapy |  |  |  |  |  | 0.1663 |
| Good | 19 | 12 | | 7 | |  |
| Poor | 11 | 3 | | 8 | |  |
| NA | 2 | 1 | | 1 | |  |
| Enneking stage |  |  |  |  |  | **0.0431** |
| Ⅰ | 6 | 5 | | 1 | |  |
| Ⅱ | 19 | 10 | | 9 | |  |
| Ⅲ | 7 | 1 | | 6 | |  |

*P*-values were calculated by Fisher's exact test or chi-square test.

Others (periosteal osteosarcoma, low-grade central osteosarcoma)

NA (Not Available)
